# The identification of functional regions of MEK1 using CRISPR tiling screens

**DOI:** 10.1038/s42003-025-07966-4

**Published:** 2025-04-24

**Authors:** Zhiqiang Zhang, Barbara Abreu, Jessica L. Brothwood, John Alexander, Martin J. Sims, John F. Lyons, Joanne M. Munck, Christopher J. Hindley

**Affiliations:** 1 Astex Pharmaceuticals, Cambridge, UK; 2 Wellcome Sanger Institute, Hinxton, UK; 3 School of Life Sciences, University of Warwick, Coventry, UK; 4 Insmed Innovation UK Ltd, Cambridge, UK

**Keywords:** Mutagenesis, Mechanism of action

## Abstract

CRISPR tiling screen is a powerful tool to identify protein regions relevant to its biological function. Understanding the functional relevance of the regions of target protein is of great help for structure-based drug discovery. Studying the drug resistance mechanisms of small-molecule inhibitors is important for the development and clinical application of the compounds. Using MEK1 and MEK inhibitors as example here, we demonstrate the utility of CRISPR tiling to identify regions essential for cancer cell viability and regions where mutations are resistant to MEK inhibitors. We study the drug resistance mechanisms of the regions and discussed the potential, as well as limitations, of applying the technology to drug development. Our findings demonstrate the value and prompt the utilization of CRISPR tiling technology in structure-based drug discovery.

## Abstract (web summary)

Utilization of CRISPR tiling screens to identify protein functional regions for structure-based drug discovery, using MEK1 as a demonstration. Biological mechanism study of distinct MEK1 drug-resistant mutations combined with structural analysis

## Introduction

CRISPR tiling is a CRISPR/Cas9-based high-throughput screening methodology, which utilises a high density of sgRNAs to tile along the coding region of a gene^1^. It was originally used for the design of sgRNAs with the best performance for gene knock-out^2^, and has since been used to identify the target protein of small-molecule compounds identified in phenotypic screens^3^. Recently, this technology has been used to identify protein functional regions (regions essential for protein functionality)^4–6^.

Fragment-based Drug Discovery (FBDD) is now recognised as an important approach to identifying ligandable sites on proteins, and together with Structure-based Drug Design (SBDD), is increasingly used to develop small-molecule compounds with therapeutic potential^7–9^. FBDD is well suited to identify potentially novel binding sites, but a challenge of this approach is that the biological (functional) relevance of the sites is sometimes unclear^10^. Furthermore, when treated with small-molecule anti-cancer drugs, tumours can acquire resistance mutations, which compromise the drug response in patients^11–13^. The mechanism leading to the drug resistance is often unclear. We recognise that CRISPR tiling could be helpful to solve these two challenges.

CRISPR tiling screens have revealed large parts of unannotated protein regions to be essential for cancer cell viability^6^. The integration of CRISPR tiling and a structural understanding of the target protein will provide valuable information on the functional relevance of the druggable sites for SBDD. Furthermore, tiling screens have been used to identify protein regions associated with drug resistance to small-molecule inhibitors^14,15^ and more recently to molecular glue degraders^16^. Further evaluation of the capacity of tiling screen to identify drug resistance mechanism is needed.

Mitogen-activated protein kinase kinase 1 (MEK1), coded by *MAP2K1* gene, is a key component of the mitogen-activated protein kinase (MAPK) pathway^17^. The structure of MEK1 has been well-studied^18,19^, and there are multiple small-molecule MEK inhibitors, which are known to bind to different sites of the protein^20–22^. Furthermore, a variety of MEK1 mutations have been identified, which confer resistance to small molecule MAPK inhibitors^23–25^. These features make MEK1 a good candidate to establish whether CRISPR tiling can be used to define the functionality of different sites and to identify the mechanisms of drug resistance.

In this paper, using MEK1 as an example, we demonstrated the potential of CRISPR tiling to identify protein functional regions required for cancer cell viability. Among the identified regions were the active and allosteric sites of the kinase, which include the target sites for small-molecule MEK inhibitors. Furthermore, a protein-protein interaction (PPI) site was confirmed to be important for the binding of MEK1 to its activator BRAF. By screening in the presence of four small-molecule MEK inhibitors, we were able to identify two regions previously not associated with drug resistance, each with a unique mechanism. These data demonstrate the feasibility of using tiling screens to identify functionally relevant sites and to define the drug resistance mechanisms.

## Results

### Drop-out screen in a parallel A375 parental line improves the detection of sgRNA depletion

Our sgRNA library consists of 300 sgRNAs in total, including non-targeting sgRNAs and sgRNAs targeting the MEK1 coding sequence (CDS) (Supplementary Table 1 and Supplementary Data 1). Optimised from a previous publication^15^, positive control sgRNAs (POS) targeting three essential genes and negative control sgRNAs (NEG) targeting four non-essential genes were added. All sgRNAs were designed to have an NGG protospacer-adjacent motif by using the CRISPick^2^ webtool.

**Fig. 1:**
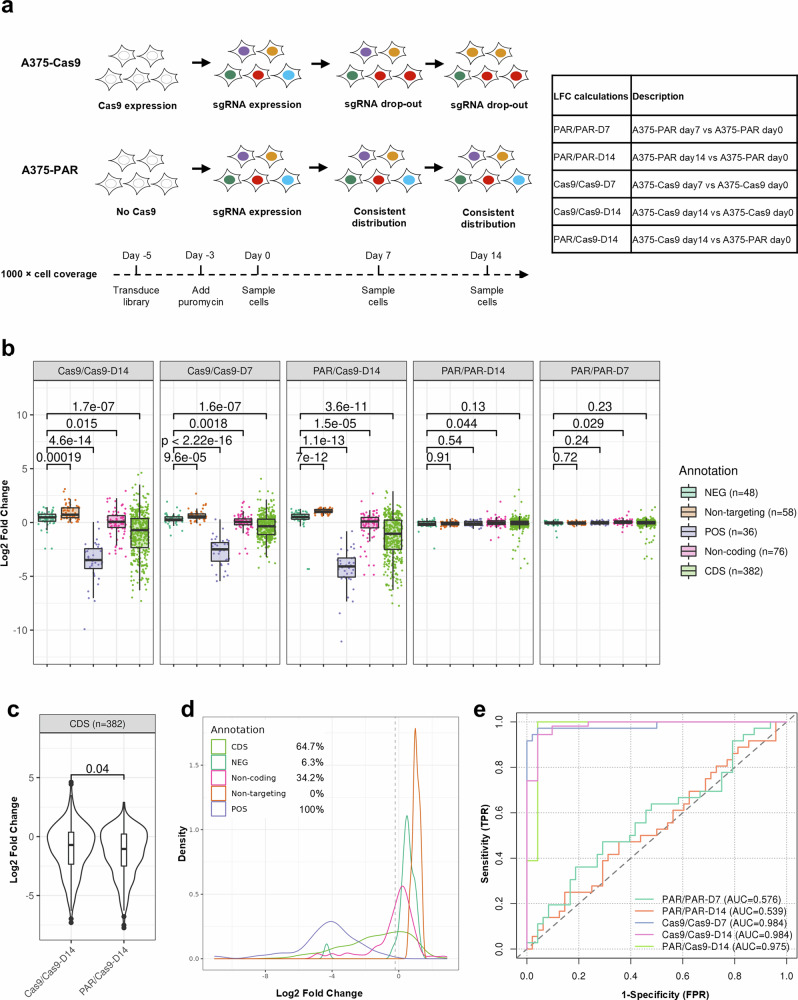
Data analysis and performance evaluation of the drop-out tiling screen. **a** A schematic protocol of the drop-out tiling screen on MEK1. A375-Cas9 and A375 parental cell lines were screened in parallel. The LFC values were calculated in different ways as indicated in the table. Different colours represent genomic integration and expression of different sgRNAs. The cell coverage and the timeline of the screen are indicated below the schematic protocol. **b** Boxplots showing the representation change of sgRNAs in different categories. Central line, median; box limits, upper (Q3) and lower (Q1) quartiles; whiskers, 1.5x interquartile range. p-values are shown above the boxes. NEG negative controls, POS positive controls, CDS MEK1 CDS-targeting sgRNAs. n = sgRNA numbers of in each category (two replicates combined). **c** Boxplot showing side-by-side comparison of MEK1 CDS-targeting sgRNAs between Cas9/Cas9-D14 and PAR/Cas9-D14 LFC values. p-value is shown above the boxes. **d** Density plots indicating the percentages of depleted sgRNAs in different categories. PAR/Cas9-D14 LFC values were plotted. Percentages of sgRNAs with LFC values less than −0.3 (dashed line) in each category are indicated. number of sgRNAs (n) in each category and the colour codes of categories are the same as in **b**. **e** Receiver operating characteristic curve (ROC) analysis of screen performance. Area under the ROC Curves (AUCs) are indicated.

A375 is a BRAF^V600E^-mutant melanoma cell line which is dependent on MEK1 (DepMap^26^) and sensitive to MEK inhibitors^24,25^. Our drop-out screen was run in A375 cells stably expressing spCas9 protein (A375-Cas9) and the parental (A375-PAR) cells in parallel (Fig. 1a). The log2 fold change (LFC) values of each sgRNA between day 7 or day 14 samples and day 0 samples for each cell line and between the cell lines were calculated and compared (Fig. 1a). For the A375-Cas9 cell line, sgRNAs targeting positive control genes were significantly depleted at day 7 and to a greater extent at day 14 (Fig. 1b). The LFC values calculated between the A375-Cas9 line at day 14 and the parental line at day 0 (PAR/Cas9-D14) showed greater significance between negative controls and MEK1 CDS-targeting sgRNAs than LFC values calculated between day 0 and day 14 of the A375-Cas9 line (Cas9/Cas9-D14) (Fig. 1b). Direct comparison also indicated that the MEK1 CDS-targeting sgRNAs were more significantly depleted in PAR/Cas9-D14, compared to Cas9/Cas9-D14 (Fig. 1c). Therefore, PAR/Cas9-D14 LFC values, which retain the maximum sgRNA depletion, were used in the following analysis.

One hundred percent of the positive control sgRNAs fell below the threshold (5th percentile of the negative controls) as depleted Fig. 1d. 64.7% of CDS-targeting sgRNAs were depleted (Fig. 1d), confirming that MEK1 functionality is essential to A375 cell viability. The computing receiver operating characteristic (ROC) curves showed effective distinction (AUC = 0.975) of the controls using PAR/Cas9-D14 LFC values (Fig. 1e). These data demonstrate the excellent performance of our screen.

### Regions at MEK1 active and allosteric sites are identified as important for cell viability by drop-out screen

**Fig. 2:**
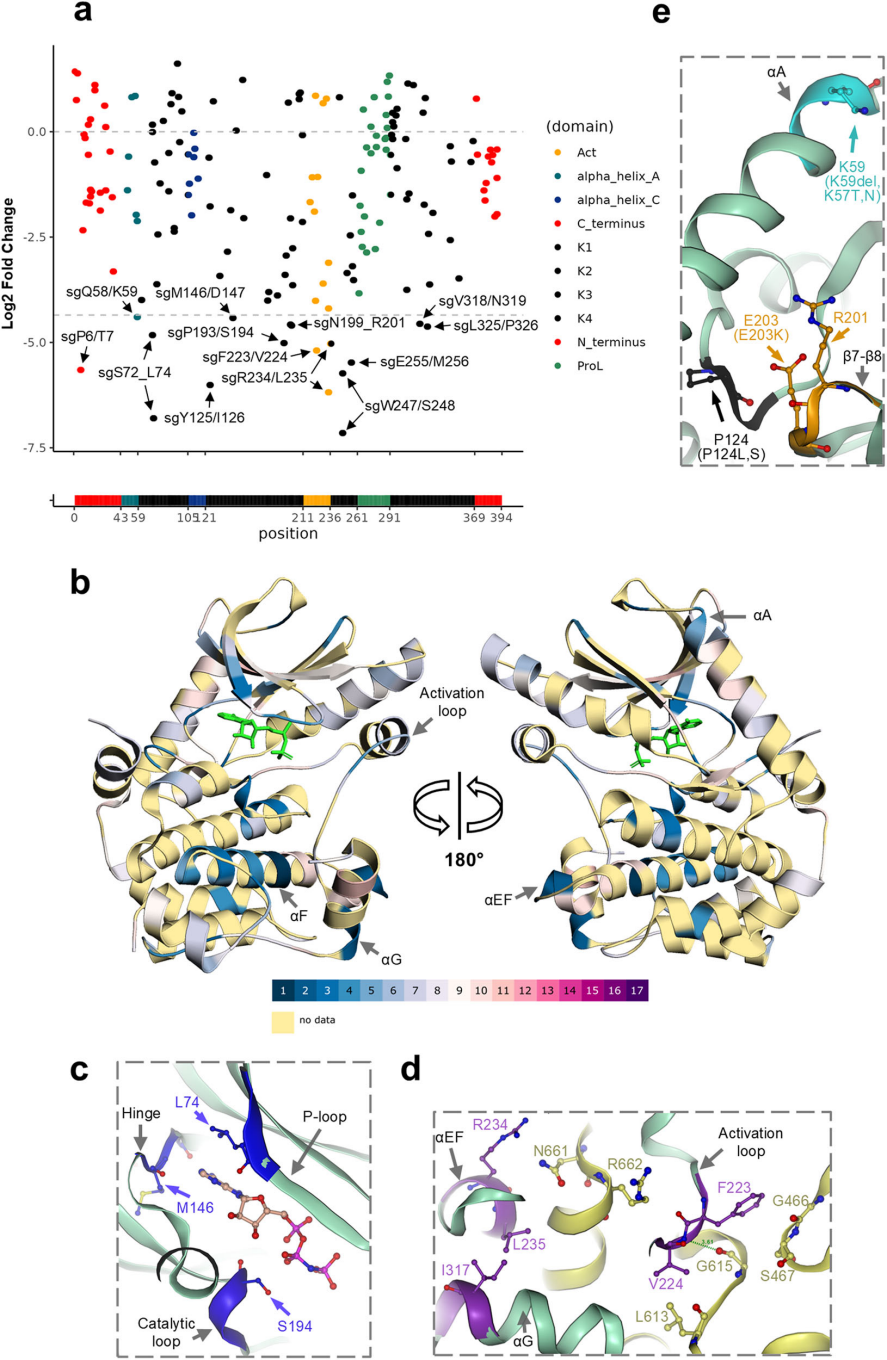
MEK1 regions were defined by drop-out screen and mapped to different protein domains. **a** PAR/Cas9-D14 LFC values were plotted to the MEK1 protein primary sequence. All sgRNAs with LFC values less than -4.35 (dashed line) are labelled as ‘sg + targeting residues’ and indicated by arrows. Dots are coloured according to MEK1 domains. A-loop: activation loop, K1-K4: core kinase domain 1-4, P-rich loop: proline-rich loop. Data points represent mean of two replicates. **b** Overview of the MEK1 regions targeted by the most depleted sgRNAs, mapped to the protein crystal structure. MEK1 residues are colour-coded by sgRNA bins in a heatmap style. Colour codes of the sgRNA bins are indicated below the structures. Molecule in green: ADP. PDB code: 3EQI. **c** A close-up view of the regions around the active site (ATP binding pocket). The molecule in the pocket is an ATP analogue, AMP-PNP. **d** A close-up view of the regions at the MEK1-BRAF PPI interface. BRAF protein ribbon is coloured in yellow. A hydrogen bond between MEK1 V224 and BRAF G615 is indicated as a dotted green line. **e** A close-up view of the regions at the MEK1 mutation hotspots. Known MEK1 oncogenic mutations at each of the regions are indicated in brackets. **c**–**e** PDB code, 6V2W. MEK1 protein ribbon is coloured in light green. MEK1 domains are labelled and indicated with arrows in grey. Key residues are displayed as ball-and-sticks, labelled, and indicated by arrows in the same colours as the highly depleted region.

The PAR/Cas9-D14 LFC values of each sgRNA were assigned to the MEK1 residues (Supplementary Data 2) and ranked into 17 bins (Supplementary Fig. 1a) using the CRISPRO computational pipeline^27^. They were plotted to the protein primary sequence (Fig. 2a) and mapped to the MEK1 3D structure in a heatmap style, reflecting the functional relevance of the targeted regions (Fig. 2b). Among the functional regions targeted by the most depleted sgRNAs (bin 16 and 17), multiple regions in the kinase active site (S72_L74, M146/D147 and P193/S194) were identified and located to the phosphate-binding (P-) loop, the hinge region, and the catalytic loop (Fig. 2c). All of these regions are known to be critical for kinase function^19^. Additionally, regions outside of the active site were also identified. Three regions (F223/V224, R234_G237 and V318/N319) were located at the MEK1 PPI interface with its upstream activator BRAF (F223/V224 also locating at the activation loop) (Fig. 2d). Moreover, three additional regions (Q58/K59, Y125/I126 and N199_R201) were located immediately next to MEK1 mutation hotspots identified in tumours^28^ (Fig. 2e). Interestingly, a region at the N-terminus around P6/T7 was targeted by one of the most depleted sgRNAs (Fig. 2a, Supplementary Data 2). This region is largely disordered and not resolved in any known crystal structures of MEK1. It has been reported that this N-terminus region is important for the binding and nuclear translocation of MEK’s direct substrate, ERK^29^.

The efficiency with which individual sgRNAs introduce mutations (on-target activity) vary^30^. To avoid causing bias on functional relevance, we corrected the drop-out screen LFC values of each sgRNA to the on-target activity predicted by CRISPRon^30^ (Supplementary Data 3). The corrected LFC values were then mapped to MEK1 protein structures (Supplementary Fig. 1b). The corrections did not change the overall identified functional regions but increased the number of highly depleted sgRNAs. For example, sgRNA targeting around G210/V211 (5′-TCTGTGACTTTGGGGTCAGC-3′) and sgRNA targeting around L92/V93 (5′-CAAGCCTTCTGGCCTGGTCA-3′) moved from bin 6 to bin 4 after the correction (Supplementary Data 3 and Supplementary Fig. 1a).

**Fig. 3:**
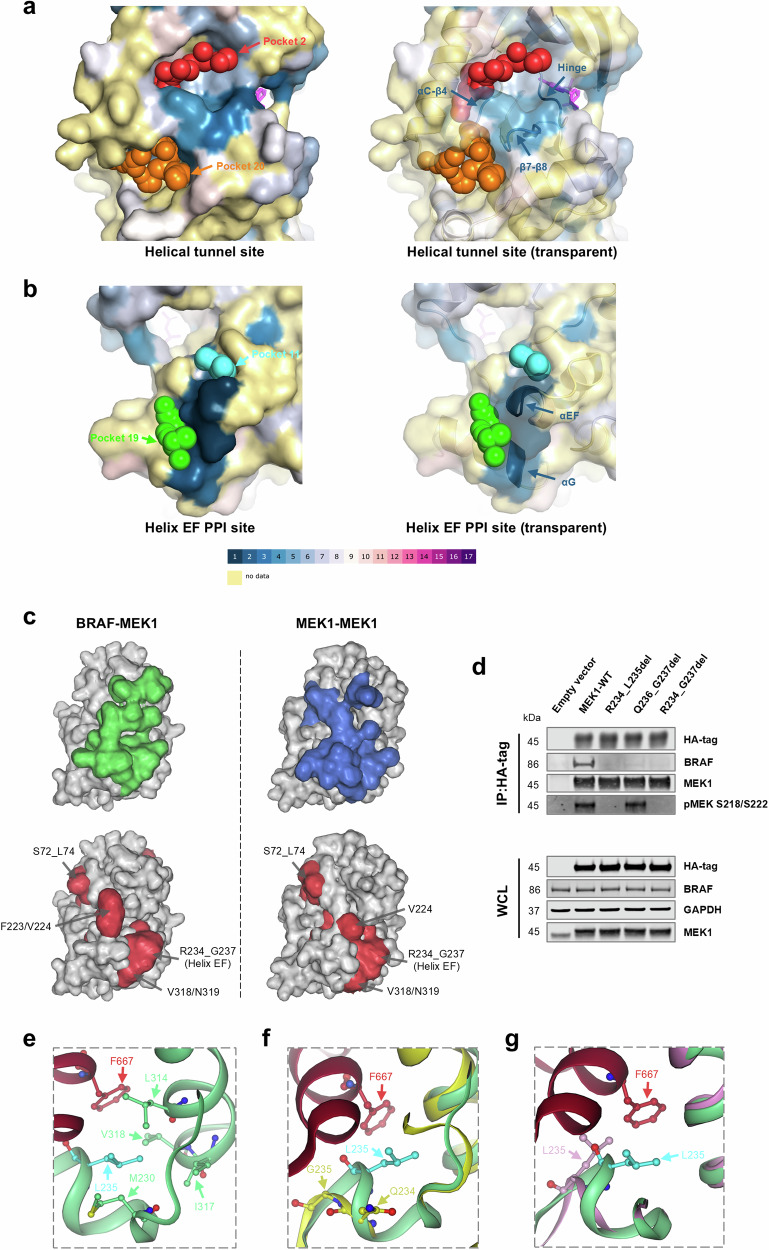
Pocket prediction, perturbation and structural analysis of identified MEK1 functional regions. Predicted pockets at the helical tunnel site (**a**) and the PPI site (**b**) are shown as coloured spheres. MEK1 surfaces (non-transparent and transparent surface) and cartoons are colour-coded by sgRNA bins in a heatmap style. Colour codes of the sgRNA bins are indicated below the structures. The predicted pockets and identified functional regions are indicated by arrows. PDB code in (**a**, **b**): 3EQI. **c** Comparison of MEK1 PPI interfaces and CRISPR tiling identified regions. MEK1 interface for BRAF binding is highlighted as surface area in green. MEK1 homodimer interface is highlighted as surface area in blue. Drop-out screen identified regions are highlighted as surface area in red and are labelled. PDB code of BRAF-MEK1 interface: 6V2W. PDB code of MEK1-MEK1 interface: 1S9J. **d** MEK1-BRAF co-immunoprecipitation. IP: immunoprecipitation. WCL: whole cell lysate. **e** A close-up view of the MEK1-BRAF interface around the region identified at Helix EF. MEK1 protein ribbon is coloured in green, BRAF in red. MEK1 L235 residue is highlighted in cyan. **f** Comparison of the MEK1-BRAF interface between MEK1 wild-type and the R234_L235del variant. MEK1 wild-type protein ribbon (PDB 6V2W) is displayed in green, R234_L235del (homology model) in yellow. **g** Comparison of the MEK1-BRAF interface between MEK1 wild-type and the Q236_G237del variant. MEK1 wild-type protein ribbon (PDB 6V2W) is displayed in green, Q236-G237del (homology model) in pink. **e**–**g** Key residues are represented by ball-and-sticks, labelled, and indicated by arrows in the same colour as the backbone.

To check whether the identified functional regions are druggable, MEK1 pockets were predicted using the computation method, Fpocket^31^. Among the predicted pockets, two of them (pocket 2 and 20) are located immediately adjacent to three identified functional loops (the hinge, β7-β8 loop and Helix C-β4 loop). These two pockets connect in the tunnel formed between Helix A and the core kinase (Fig. 3a). Another two pockets (pocket 11 and 19) are located at the identified functional relevant PPI site (Helix EF and Helix G) (Fig. 3b). However, apart from the active site and the well-known allosteric site next to it (pocket 1), all other pockets have low druggability scores (>0.5 is typically classified as ‘druggable’^32^) (Supplementary Data 4). This may explain why no compounds have been developed to target MEK1 at any other pockets, apart from the active and allosteric sites.

### The identified region at MEK1 Helix EF is required for MEK1-BRAF binding

There are a set of common MEK1 residues between the interfaces of the BRAF-MEK1 heterodimer and the MEK1-MEK1 homodimer^33^. Among the identified regions described above, four regions (S72_L74, F223/V224, R234_G237 and V318/N319) clustered at the MEK1 PPI interface (analysed by PDBePISA^34^) (Fig. 3c). Two small molecules binding at the allosteric site have previously been shown to extend to the PPI site in structures of MEK1 complexes^22,35^. These crystal structures showed that trametinib and avutometinib form polar interactions with MEK1 residue R234 (Supplementary Fig. 2a). In order to validate the functional relevance of the R234_G237 region, we assessed the mutations at this single locus generated by sgR234/L235. sgR234/L235 was transduced into A375-Cas9 cells to generate mutations, the representations of which were monitored by NGS sequencing over 9 days. LFC values of each mutation were calculated and ranked as most and least depleted (Supplementary Data 5). Small deletions spanning R234 to T238 were depleted to the highest extent, similar to the three most abundant frameshift mutations (Supplementary Table 2). In contrast, the wild-type and several single residue substitutions (e.g., G237V) or synonymous mutations (L235=) were enriched or depleted to a lesser degree (Supplementary Table 3). We also compared the overall depletion of frameshift versus in-frame mutations among all insertion-deletion (INDEL) mutations generated by sgR234/L235 across 3 time points (Supplementary Data 6). Our results showed that frameshift and in-frame mutations were depleted in a similar way within the cell population (Supplementary Fig. 2b), in agreement with the behaviour of sgRNAs targeting protein functional regions^4^. These results showed that perturbations at this specific region affect A375 cell viability, comparable to the knock-out of the gene.

Due to its location at the PPI interface, the importance of this region on MEK1-BRAF binding was tested. We expressed HA-tagged MEK1 small deletion variants (R234_L235del, Q236_G237del and R234_G237del) in cells and determined their binding with endogenous BRAF by co-immunoprecipitation (co-IP). All three variants abolished MEK1-BRAF binding, despite having the same expression levels as the wild-type MEK1 (Fig. 3d). Interestingly, the Q236_G237del variant remained phosphorylated, despite the abolition of BRAF binding (Fig. 3d). This is not likely to be caused by a change of preference on binding of RAF isoform, as there is no obvious difference observed on their binding with CRAF or ARAF (Supplementary Fig. 2c).

Analysis of the MEK1 crystal structure revealed that the loop containing residues R234_T238 is directly involved in the contact interface with BRAF (Fig. 3e). L235 is deeply buried in the interface, and close to a cluster of hydrophobic residues that include MEK1 L314, I317, V318, M230 and BRAF F667 (Fig. 3e). Our single locus editing described above showed that several of the most depleted variants contained the L235 deletion (Supplementary Table 2), indicating its importance to MEK1 function. Hydrophobic residues at the protein interfaces are important for the stabilisation of protein complexes^36^. In the homology model (obtained using AlphaFold^37^) of the MEK1 R234_L235del variant, this loop is shortened compared to the wild-type (Fig. 3f) (Supplementary Data 9 and 10). Homology model of another MEK1 variant, Q236_G237del, showed a displacement of L235 away from the hydrophobic cluster (Fig. 3g) (Supplementary Data 11). The residues deleted in both variants possibly hindered the integrity of the hydrophobic cluster. In contrast, the G237V homology model showed marginal structure changes in residue T238 and H239 orientation (Supplementary Fig. 2d) (Supplementary Data 12). These residues do not contribute to the PPI, hence the changes are unlikely to affect MEK1-BRAF binding. This explains why G237V was enriched in the single locus editing, similar to MEK1 wild-type (Supplementary Table 3). Our structural analysis further suggests that some mutations introduced at the identified region affect MEK1 function, most likely by influencing MEK1-BRAF binding.

### Enrichment screens with a panel of MEK inhibitors identify additional functional regions for drug resistance

**Fig. 4:**
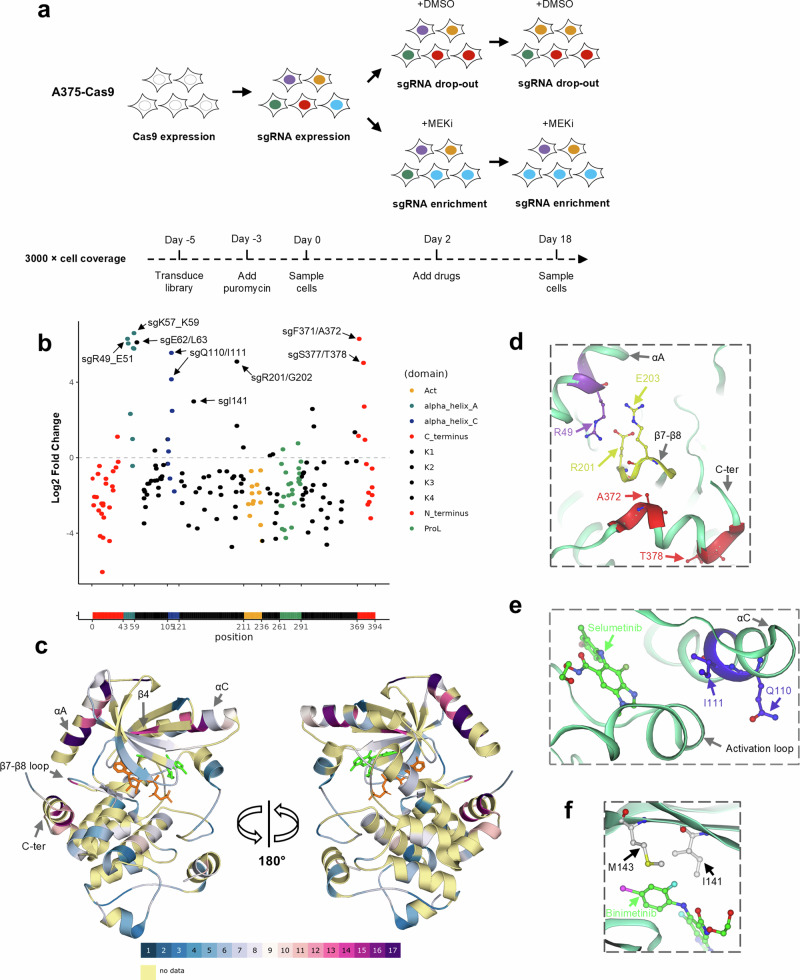
MEK1 regions were defined by enrichment screens and mapped to different protein domains. **a** A schematic protocol of the tiling enrichment screens on MEK1. A375-Cas9 cell line were transduced with sgRNA library and treated with DMSO or one of the MEK inhibitors: selumetinib, trametinib, cobimetinib or binimetinib. The LFC values of each sgRNA between the drug treatment arm and DMSO arm at day 18 was determined. Different colours represent genomic integration and expression of different sgRNAs. The cell coverage and the timeline of the screens are indicated below the schematic protocol. **b** LFC values of each sgRNA in each screen were plotted to the MEK1 protein primary sequence. The most enriched sgRNAs are labelled as ‘sg + targeting residues’ and indicated by arrows for the binimetinib screen. Dots are coloured according to MEK1 domains. A-loop activation loop, K1-K4 core kinase domain 1–4, P-rich loop proline-rich loop. Data points represent mean of two replicates. **c** Overview of the MEK1 regions targeted by the most enriched sgRNAs, mapped to the protein crystal structure. MEK1 residues are colour-coded by sgRNA bins in a heatmap style. Colour codes of the sgRNA bins are indicated below the structures. Molecule in green: AMP-PNP, molecule in orange: binimetinib. **d** A close-up view of regions at Helix A, the β7-β8 loop and the C-terminus. **e** A close-up view of a region at MEK1 Helix C domain. **f** A close-up view of a region at the allosteric drug binding pocket. The highlighted residue M143 is the gatekeeper of the ATP binding pocket. **c**–**f** MEK1 protein ribbon is coloured in light green. MEK1 domains are labelled and indicated with arrows in grey. Key residues are displayed as ball-and-sticks, labelled and indicated by arrows in the same colours as the highly enriched region. PDB code for structures in (**d**, **e**) 7JUZ, in (**c**, **f**) 7M0U.

In order to evaluate whether CRISPR tiling could identify regions associated with resistance to different MEK inhibitors, a panel of screens with selumetinib, trametinib, cobimetinib and binimetinib were carried out in parallel at concentrations equivalent to the IC_50_s of the parental A375 line (43 nM, 2 nM, 2.2 nM and 24 nM, respectively) (Fig. 4a). sgRNA LFC values were calculated between drug treatment and DMSO treatment at day 18 and plotted to the MEK1 primary sequence (Supplementary Data 7). Among the most enriched sgRNAs (bin 1 and 2 out of 17 bins, LFC > 4.16) in the selumetinib screen were a cluster of sgRNAs targeting MEK1 N-terminus (R49_E51, K57_K59 and E62/L63) regions, together with two sgRNAs targeting Q110/I111 at Helix C, in agreement with a previous study^15^. Surprisingly, sgRNAs targeting the very C-terminus of MEK1 (around F371/A372 and S377/T378) were observed to be highly enriched (Supplementary Fig. 3a). Comparing to the screens with other drugs, the N-terminus and Q110/I111 regions were identified in all four screens, while the C-terminus region was identified in the screens against three out of four MEK inhibitors (Fig. 4b and Supplementary Fig. 3a–c), the exception being trametinib, which is known to have a unique binding mode^22^. Moreover, a sgRNA targeting around R201/G202 was enriched in all four screens, despite a comparably moderate enrichment in the screen with trametinib (LFC = 3.3) (Fig. 4b and Supplementary Fig. 3a–c) (Supplementary Data 7).

When mapped to the MEK1 crystal structure, all of the above regions resided within domains distal from the active site (Fig. 4c). The regions around R49_E51 and K57_K59 mapped to Helix A (Fig. 4d), which is the negative regulatory domain for MEK1 kinase activity^38^. The other region around R201/G202 mapped to the β7-β8 loop, which is juxtaposed to and interacts with Helix A (Fig. 4c, d). Both regions cluster into known oncogenic MEK1 mutation hotspots^28^. MEK1 mutations (F53C, Q56P, K57T, K59del, E203K), which have been identified in patients and demonstrated to confer resistance to MAPK inhibitors^25,39,40^, cluster into these two regions. The region around Q110/I111 mapped to Helix C, which is adjacent to the allosteric pocket for compound binding (Fig. 4e). MEK1 mutations clustered into this region (I111N and L115P) have also been reported to confer drug resistance^25,41^.

The C-terminus regions around F371/A372 and S377/T378 were located just upstream of the highly disordered end of MEK1 (T386_V393) (Fig. 4d). Mutations clustered at this C-terminus region have been identified in tumours (GENIE dataset v10^42^) (Supplementary Table 4), and a N382H mutant is classified as a pathogenic mutation, leading to melanoma (ClinVar^43^) (Supplementary Table 5). However, this region has not been reported to be associated with drug resistance. An E368K mutation was previously identified in MEK inhibitor-resistant clones generated by a mutagenesis scan without validation^24^.

Interestingly, one sgRNA targeting around I141/C142 was moderately enriched only in the screen with binimetinib (LFC = 3.1) (Fig. 4b and Supplementary Data 7). Mapping to the protein structure, it reveals that this region lies within the allosteric pocket where the MEK inhibitors bind (Fig. 4F). Structurally, this region is unlikely to be binimetinib-specific, as it interacts with the arylamine group, which is common to all compounds used in our screens^35^. Mutations at this region are very rarely identified in tumours (GENIE dataset v10) (Supplementary Table 4) and a M143V variant is classified as likely pathogenetic (ClinVar) (Supplementary Table 5). There is no reported association between this region and drug resistance.

### sgRNAs targeting the identified regions can generate drug resistant clones in two cancer cell lines

Five sgRNAs (Supplementary Table 6) targeting each of the regions identified by the enrichment screens (sgQ58/K59, sgQ110/I111, sgI141/C142, sgR201/G202 and sgF371/A372) were validated individually. Although only moderately enriched in the screen with binimetinib, sgI141/C142 was included. The ability of these sgRNAs to generate resistance clones was tested in A375-Cas9 cells by single locus editing and selection with 0.1 µM binimetinib. The emergent drug-resistant single cell clones were genotyped by NGS sequencing. As expected, all the genotyped clones were found to contain at least one allele of an in-frame MEK1 mutation, indicating the association between drug resistance and mutations at each identified region. The in-frame mutations identified among the clones were aligned to the gene sequence of wild-type MEK1. Considering the mutation types in the resistant clones, sgQ58/K59, sgQ110/I111 and sgF371/A372 generated mostly nucleotide deletions, with two insertions generated by sgQ58/K59. Differently, sgI141/C142 and sgR201/G202 generated nucleotide substitutions as majority. At the C-terminus region, recurrent mutations coding A372del and G373del variants were observed among the resistant clones generated by sgF371/A372 (Supplementary Fig. 4a). Deep sequencing of the pooled drug-resistant population showed good consistency with the genotyped single cell clones (Supplementary Fig. 5). However, why these mutations would cause drug resistance is unknown.

The most enriched variants at each region, including K59del, I111del, I141_C142delinsEI, G202_E203delinsEL and A372del, were selected as representatives of each site for further investigation (Supplementary Table 7). Drug resistance of these selected mutations was verified by CRISPR knock-in with and without homology-directed repair (HDR) donor template. Presence of HDR donor significantly increased cell viability in response to binimetinib, compared to using sgRNA alone (Supplementary Fig. 4b). Adding an HDR enhancer, to increase the knock-in efficiency, further increased cell viability (Supplementary Fig. 4b). These results indicate that the selected mutations are indeed the cause of resistance, rather than random edits by the sgRNA alone.

Three of these variants (K59del, I141_C142delinsEI and A372del) were introduced into the endogenous MEK1 in a further BRAF^V600E^-mutant cell line, HT-29, by CRISPR knock-in. The three CRISPR edited cell pools were selected with 5 nM trametinib, and the representations of the MEK1 mutations were assessed by NGS sequencing. As the most abundant mutations in each of the cell pools, correct knock-ins of K59del, I141_C142delinsEI and A372del were detected in 29%, 42% and 40% of all MEK1 alleles, respectively (Supplementary Fig. 6a). This further confirms the association between all three mutations and the resistance to trametinib. Surprisingly, A372del was enriched by trametinib selection here, despite the targeting region not being identified in the tiling screen with trametinib. Positive single cell clones with at least one allele of each correct knock-in mutation were selected and hereafter referred to as HT29-K59del, HT29-I141_C142delinsEI and HT29-A372del cell lines.

### The resistance mutations either hyperactivate the MAPK pathway or perturb the compound binding site

**Fig. 5:**
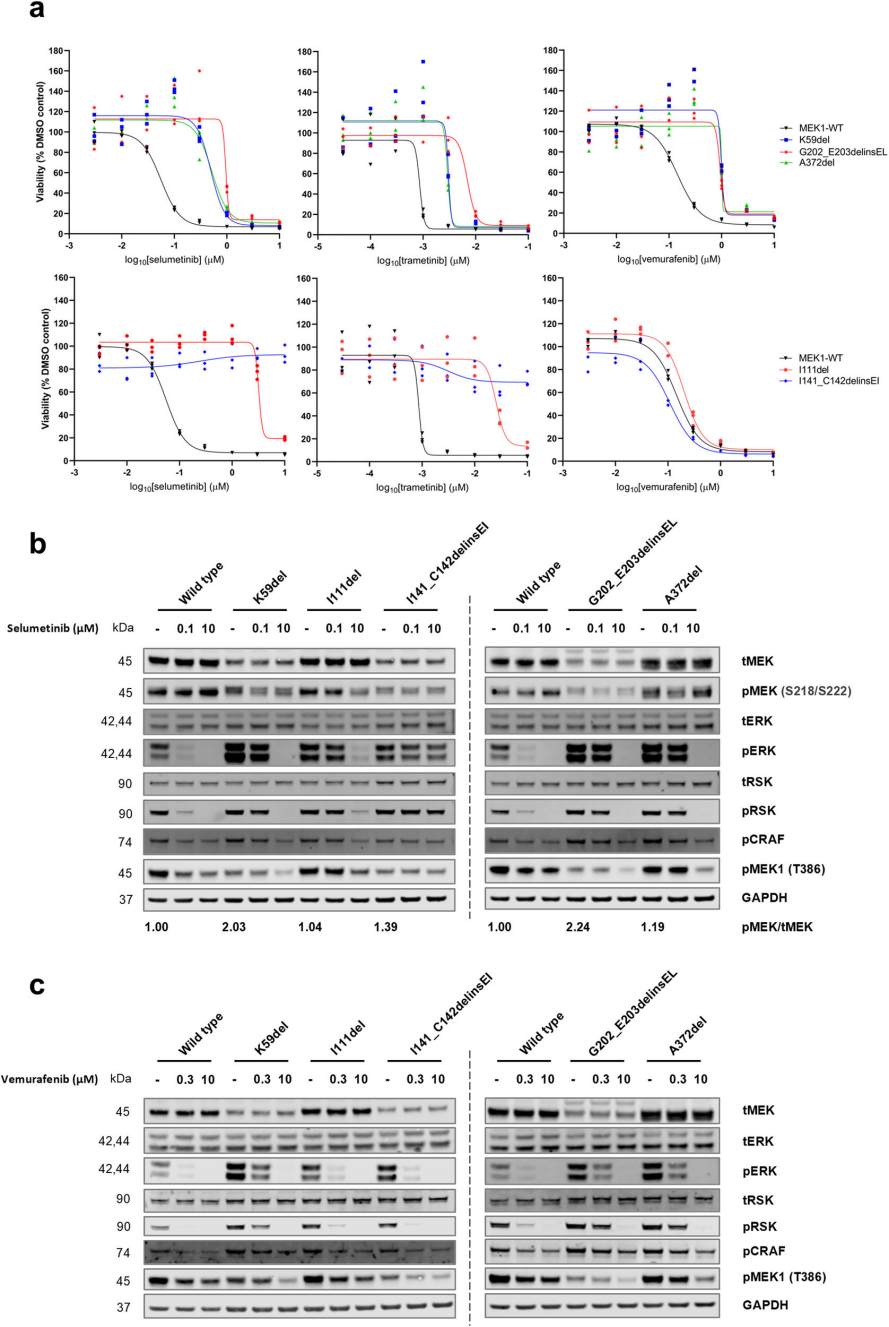
MEK1 mutations at the identified regions had different sensitivities to MEK and BRAF inhibitors in cell viability and MAPK pathway signalling assays. **a** Dose response curves of A375 cell line viability in response to selumetinib, trametinib or vemurafenib. The upper panel of curves include the MEK1 activating mutations and the lower panel include the allosteric pocket influencing mutations. Cell viability is shown as percentage to the DMSO control. Data points with three replicates are shown. **b** MAPK pathway signalling upon selumetinib treatment. Effects of 2 h treatment with 0.1 µM or 10 µM selumetinib on MAPK pathway signalling were tested in the A375 cell lines. **c** MAPK pathway signalling upon vemurafenib treatment. Effects of 2 h treatment with 0.3 µM or 10 µM vemurafenib on MAPK pathway signalling were tested in the A375 cell lines. DMSO treatment is indicated as ‘-’. Band intensity was quantitated by densitometry. And fold changes of pMEK (S218/S222)/tMEK ratios to the wild-type are indicated below the plots as numbers.

To elucidate the drug resistance mechanism of the mutations, we generated isogenic pairs of A375 cells with the selected variants at each site (K59del, I111del, I141_C142delinsEI, G202_E203delinsEL and A372del) by CRISPR knock-in. Hereafter, these cell lines are termed A375-K59del, A375-I111del, A375-I141_C142delinsEI, A375-G202_E203delinsEL and A375-A372del, respectively. Sensitivity of the cell lines to MEK inhibitors (selumetinib and trametinib) and a BRAF inhibitor (vemurafenib) were determined in a viability assay. The A375-K59del, A375-I111del, A375-G202_E203delinsEL and A375-A372del cell lines all showed significantly reduced sensitivity to selumetinib (ranging from 8.93- to 57.8-fold change in IC_50_ values compared to the parental line), whilst A375-I141_C142delinsEI was not sensitive to any tested concentration (Fig. 5a). A similar pattern was observed in response to trametinib, although with smaller but significant shifts in IC_50_ values (Fig. 5a). Moreover, A375-K59del, A375-G202_E203delinsEL and A375-A372del showed significantly reduced sensitivity to vemurafenib, whilst A375-I111del and A375-I141_C142delinsEI remained sensitive, with no significant difference compared to the parental line (Fig. 5a).

Drug sensitivity was also tested in the HT29 cell lines knocked-in with the MEK1 mutations. Interestingly, HT29-K59del and HT29-A372del cell lines, but not HT29-I141_C142delinsEI, showed significantly higher basal metabolism, compared to the parental line (Supplementary Fig. 6b). Proliferation of the cell lines upon drug treatment was therefore measured by cell confluency. As expected, loss of sensitivity to both selumetinib and vemurafenib was observed for HT29-K59del and HT29-A372del, whereas HT29-I141_C142delinsEI was sensitive to vemurafenib but not selumetinib (Supplementary Fig. 6c, d). The data above confirm that mutations at the identified regions cause drug resistance, but with different sensitivities to MEK and BRAF inhibitors.

We then determined the effects of selumetinib on MAPK signalling in the A375 cell lines. The A375-K59del, A375-G202_E203delinsEL and A375-A372del cell lines displayed pathway hyperactivation (high basal phospho-ERK compared to parental cells), in the absence of compound (Fig. 5b). They had reduced sensitivity to 0.1 µM selumetinib (high phospho-ERK and high phospho-RSK) but remained sensitive to 10 µM selumetinib (Fig. 5b). A375-I111del also displayed a moderate increase in basal pathway signalling and reduced sensitivity to selumetinib (Fig. 5b). A375-I141_C142delinsEI had similar basal pathway activity to the parental line but lost sensitivity to selumetinib at both concentrations tested (Fig. 5b), potentially suggesting a steric hindrance to selumetinib binding. The pathway signalling was also characterised in the presence of vemurafenib. As was the case with selumetinib, A375-K59del, A375-G202_E203delinsEL and A375-A372del showed high basal pathway signalling and reduced sensitivity to vemurafenib (Fig. 5c). In contrast, MAPK signalling of A375-I111del and A375-I141_C142delinsEI cell lines was inhibited by vemurafenib (Fig. 5c). Our MAPK signalling assays indicate two resistance mechanisms: K59del, G202_E203delinsEL and A372del are likely to be MEK1 activating variants causing pathway hyperactivation, while I111del and I141_C142delinsEI are variants likely to perturb MEK inhibitor binding. Some MEK1 mutations, which cluster at the same regions as our mutations, have been reported to share the same resistance mechanisms and show the same effects on sensitivity of MEK and BRAF inhibitors^25^.

### Structural analysis of MEK1 I141_C142delinsEI and A372del variants provides insight into mechanisms of drug resistance

**Fig. 6:**
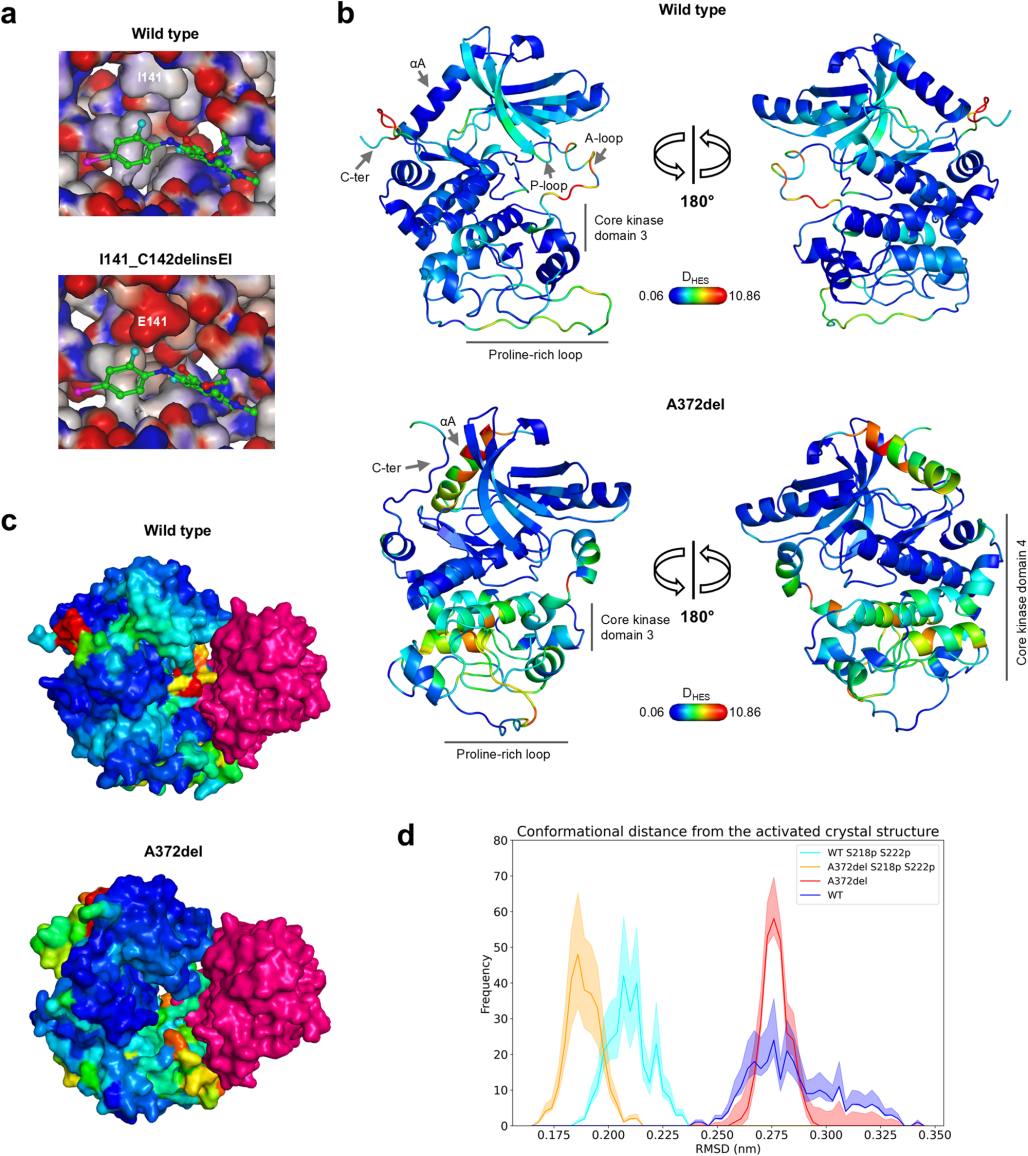
Structural analysis demonstrated that I141_C142delinsEI causes steric hindrance while A372del potentially alters conformation at the PPI interface. **a** Mapping electrostatic potentials to the inhibitor binding pocket. Both structures of the MEK1 wild-type and I141_C142delinsEI variant are homology models. The small molecule binimetinib is superimposed from the MEK1 crystal structure (PDB code: 7M0U). Surface area of the pocket was coloured by electrostatic potentials in red (negatively charged) or blue (positively charged). The residues at position 141 are labelled. **b** Conformational changes between the di-phosphorylated and non-phosphorylated forms of simulated MEK1 wild-type and A372del ensembles by MD. The structures are the mean of the representative conformations of the di-phosphorylated forms. The colours mapped on the structures correspond to the D_HES_ values. The higher the D_HES_ value, the higher the magnitude of the conformational change. The dark blue colours correspond to the minimum observed values. **c** The conformational changes, represented by D_HES_, were mapped to the crystal structure of the MEK1-BRAF complex (PDB code: 7M0Y). The same colouring was used as in **b**. **d** Conformational difference between each of the simulated ensemble and the active MEK1 crystal structure. C-alpha RMSD distributions were obtained by comparing the trajectories of each ensemble with the S218D/S222D double mutant MEK1 crystal structure (PDB code: 4ANB). Residues across the whole MEK1 protein were compared. Mean of RMSD from three replicates is indicated in solid line. The shades represent error with 95% confidence interval obtained with bootstrapping.

Next, we sought to understand the drug resistance mechanisms of MEK1 I141_C142delinsEI and A372del variants in terms of their impact on the protein structure. For the I141_C142delinsEI variant, our AlphaFold homology models implied that the isoleucine to glutamate substitution at position 141 would cause not only steric hindrance with binimetinib due to increased sidechain length but also a change in protein electrostatic potential, resulting in an unfavourable interaction with electron-rich moieties on the drug (Fig. 6a) (Supplementary Data 13). In contrast, residue C142 is orientated away from the binding site and its vicinity is populated by hydrophobic residues such as F133, F68, L63 and Y130. Hence, its replacement by an isoleucine may not have a profound structural impact.

In contrast to the I141_C142delinsEI variant, the impact of the A372del mutation on the MEK1 structure is not immediately clear from the homology models (Supplementary Data 14). We therefore speculated that flexibility could play a role and implemented molecular dynamics (MD) simulations on both the MEK1 wild-type and the A372del variant, to gain an insight into the structural effects.

Firstly, we compared the conformational changes between non-phosphorylated (inactive) and S218/S222 di-phosphorylated (active) forms of both the wild-type and A372del ensembles. Reflected by the harmonic ensemble similarity (D_HES_), conformational changes between the di-phosphorylated and non-phosphorylated ensembles of the wild-type MEK1 (di-pho-WT and non-pho-WT) were observed across the protein in the P-loop, the activation loop, the hinge region, the core kinase domain 3 (CKD3), a segment of the proline-rich loop and the C-terminus (Fig. 6b and Supplementary Fig. 7a). The representative conformations of each state showed that the P-loop moves towards the front of the active site and the hinge motif tends to adopt a more rigid conformation (Supplementary Fig. 8a). In contrast, the conformational changes between the di-phosphorylated and non-phosphorylated ensembles of the A372del variant (di-pho-A372del and non-pho-A372del) showed some difference. The conformational changes were observed in the CKD3, a portion of the proline-rich loop and the CKD4. Interestingly, a large conformational change was observed at the N-terminus, where the negative regulatory Helix A lies (Fig. 6B and Supplementary Fig. 7a), suggesting an effect on MEK1 kinase activity. Different representative conformations were observed in a segment of the CKD4 (residues 307 to 318) (Supplementary Fig. 8b), a region involved in the protein interaction (Fig. 6c). This implies that the A372del variant may influence the binding of BRAF.

Next, to assess the potential physiological relevance of the A372del variant, the conformations of the MD ensembles were compared with a crystal structure of an active S218D/S222D double-mutated MEK1 bound to BRAF. As shown by the C-alpha root-mean-square deviation (RMSD) distributions, both the di-pho-WT and di-pho-A372del ensembles showed closer conformations to the active crystal structure, compared to non-pho-WT and non-pho-A372del (Fig. 6d). Interestingly, the di-pho-A372del ensemble, compared to di-pho-WT, is conformationally closer to the crystal structure when comparing residues across the whole protein (Fig. 6d) or residues only at the PPI site (Supplementary Fig. 7b). Based on these observations, we implicitly assume that the A372del is an MEK1 activating mutation possibly caused by the changes on BRAF binding.

## Discussion

In this study, we described for the first time of using paired cell lines (Cas9^+^ and Cas9^-^) to improve the performance of a CRISPR tiling drop-out screen. We confirmed one of the identified regions at the PPI interface to be essential for MEK1-BRAF protein interaction. Moreover, drug resistance regions and mutations (Supplementary Table 7) to MEK and BRAF inhibitors were identified and classified by our mechanistic studies and structural analysis.

CRISPR tiling data is useful to define the biological functionality of the fragment binding sites in structure-based drug discovery. Two sites identified in our CRISPR-tiling screen were previously identified in fragment-screens against MEK1: E144 located at the hinge region^44,45^ and S212 at the activation loop^46^. Effective small-molecule MEK ATP-competitive inhibitors (E6201, DS03090629, BI847325) and allosteric inhibitors (trametinib, binimetinib, avutometinib), which interact with these sites (PDB: 5HZE, 7XLP, 5EYM, 7JUR, 7M0U, 7M0Z) have been developed, thus confirming their functional relevance. Combined with the functional data of CRISPR tiling, hits from the fragment-screens could potentially be prioritised based on the functional relevance of the targeting region, thus streamlining the drug discovery process. It will be interesting to have more extensive comparisons between FBDD and tiling data of more targets. Furthermore, the other functional regions identified here, which have not been targeted by any small-molecule compounds, represent potential opportunities for drug development that could be explored further. However, the predicted poor druggability scores across MEK1 pockets may explain why the pocket where the active and allosteric sites are located is the only one being targeted by compounds so far.

CRISPR tiling data is also potentially useful to guide the design of small-molecule compounds. Many type III MEK allosteric inhibitors (e.g., selumetinib, trametinib, binimetinib, avutometinib) share a conserved binding pocket, interacting with K97, V127 and S212 (PDB 7JUZ, 7JUR, 7M0U, 7M0Z). High depletion of sgRNAs targeting regions near these residues (L92/V93, Y125/I126 and G210/V211) was observed in our drop-out tiling screen. The importance of interaction with one of the catalytic residues K97 and the activation loop at S212 has been emphasised on the design of MEK allosteric inhibitors, because they are critical for the kinase activity^47^. However, the functional relevance of compounds’ interaction with V127 has potentially been overlooked. Our data show that region at the αC-β4 loop, where V127 locates, is functionally important. This is supported by P124, at the same region, being one of the oncogenic mutation hotspots^28^. Hence, the interaction with V127 potentially contribute not only to the compound binding but also to the disruption of MEK1 function. Moreover, another MEK1 oncogenic hotspot at the β7-β8 loop^28^ was defined as a functional region (around R201/G202) by our screen. This region has not been targeted by any compound and remains open to development. As the two regions above are close to each other, it might be advantageous to elaborate the current V127 binding compound towards the β7-β8 loop for a potentially better inhibition effect, if structurally possible.

In addition to the conserved binding pocket, some MEK allosteric inhibitors bind to MEK1 at the catalytic loop (avutometinib, cobimetinib) or at residue R234 (avutometinib, trametinib) (PDB 7M0Z, 7JUY, 7JUR). Both regions were identified by our tiling screen. Compound interaction with the catalytic loop is straightforward on inhibiting kinase activity. However, the functional significance of compound binding to R234 has not been elucidated. Our data show that the disruption of this region completely abolishes BRAF-MEK1 binding, supporting its importance in binding and phosphorylating ERK^33^. Hence, the compounds might be benefited by molecular interaction with R234 on MEK inhibition.

There are limitations to the use of drop-out screens to identify functional regions. sgRNA on-target activities and frameshift mutation rates vary, depending on the genome binding sequence^30,48,49^. In tiling screens, LFC values of sgRNAs targeting different regions of a protein are compared. Both the sgRNA on-target activity and frameshift mutation rate will introduce bias on defining the functional regions. Prediction tools for sgRNA activity and edit outcome are both available^30,48,49^, which have not yet been used in tiling data analysis. Here, we simply corrected the sgRNA LFC values to predicted on-target activity and frameshift rate. The corrections changed the extent of depletion of certain sgRNAs, but not the overall identified functional regions. Another limitation of this study is that the use of NGG PAM restricts the sgRNA density (47.5% MEK1 residues covered on average) (Supplementary Data 15). Using PAM-flexible Cas9 nucleases^50–52^ will improve the coverage and hopefully increase the sensitivity for region identification.

CRISPR tiling data is useful to address drug resistance mechanisms. MEK1 mutations have been known to cause resistance to MAPK inhibitors by two drug resistance mechanisms: destabilising the inhibitory Helix A, which leads to the activation of MEK1, or disruption of the allosteric pocket, which hinders MEK inhibitor binding^24,25^. The MEK1 regions identified by our enrichment tiling screens cover both resistance mechanisms. Two of the identified regions have not previously been reported to be associated with drug resistance. We studied the resistance mechanisms associated with these regions, with the support of structural analysis and classified them to each of the known mechanisms. This demonstrates the utility of CRISPR tiling screens to study drug resistance mechanisms. The information is also potentially useful to predict drug response in patients and to develop differentiated drugs. Unlike the mutations generated by Cas9 in our study, the drug resistance mutations identified in patients are most commonly single nucleotide variants (SNVs). However, these clinical mutations cluster at the same regions of MEK1 and share the same resistance mechanisms as the regions identified by tiling screen. This demonstrates the feasibility of using tiling screens to identify resistance regions and mechanisms, rather than the usage to classify single SNVs. MEK1 mutation and resistance to MEK inhibitors have been well-studied, and it will be interesting to apply the approach further to other targets, e.g., KRAS and the more recently developed KRAS G12C inhibitors.

In conclusion, CRISPR tiling technology, combined with structural analysis, can define the biological relevance of protein regions bound by fragments or small-molecule compounds. The approach can also be used to uncover drug resistance regions, as well as mechanisms. We applied the method to MEK1, mapping its functional regions and those that play a role in MAPK inhibitor resistance. Our results demonstrate the value of utilising the approach in structure-based drug discovery.

## Material and methods

### Mammalian cell culture

The A375 cell line was obtained from American Type Culture Collection (ATCC). The HT-29 and HEK293 cell lines were obtained from The European Collection of Authenticated Cell Cultures (ECACC). Cells were cultured according to supplier’s recommendations. A375 and HT-29 cell lines with MEK1 mutations constructed with CRISPR knock-in were maintained in medium with 0.1 µM binimetinib and 3 nM trametinib, respectively. All culture media and supplements were purchased from Thermo Fisher (Gibco products, Paisley, UK). All cell lines were not passaged for more than 6 months (or 30 passages) after authentication by the cell bank (short tandem repeat PCR) and were routinely screened for mycoplasma (MycoAlert, LONZA, Slough, UK). The identity of each cell line was routinely validated by the presence of unique genetic modifications to ensure cross-contamination did not occur.

### A375-Cas9 cell line construction

A375-Cas9 cells were constructed using Edit-R^TM^ Lentiviral Cas9 Nuclease (Horizon Discovery, VCAS10126). 5E4 cells were seeded in 24-well plates and transduced with the lentiviral Cas9 particles at MOI 0.3 and 0.5 in 500 µL FBS-free media with 5 µg/mL hexadimethrine bromide (polybrene) (Merck). Cells were selected with 12.5 µg/mL Blasticidin S HCl (Thermo Fisher). Single cell clones were isolated by BD FACSMelody™ Cell Sorter (BD Biosciences) in 96-well TC plates. Single cell clones Cas9 expression levels were detected by western blotting using anti-Cas9 primary antibody (Supplementary Table 8).

### Lentiviral sgRNA library production (Thermo Fisher scientific)

The sgRNA library for MEK1 tiling screens was designed in-house and was produced by Thermo Fisher as Invitrogen™ LentiArray™ CRISPR gRNA Lentivirus. The positive and negative genes were randomly selected according to DepMap dependency scores (Chronos dataset)^26^ in the A375 cell line (positive controls with dependency <−2 and negative controls with dependency around 0). Six sgRNAs targeting each of these control genes were selected from one of the genome-wide screen libraries to ensure good efficiency^2,53^. The sgRNAs were cloned into a LentiPool CRISPR Library and then packaged into lentiviral particles as a pool.

### CRISPR/Cas9 tiling screens and next generation sequencing (NGS)

A375 parental or A375-Cas9 cells were infected by the lentiviral sgRNA library at MOI 0.3 in FBS-free media with 5 µg/mL hexadimethrine bromide (polybrene) (Merck) for 2 days. Cells were then selected with 2 µg/mL puromycin dihydrochloride (Thermo Fisher) for 3 days. The day 0 cells were sampled and split into different arms. Each arm of the screen was run in two replicates with cell coverage of at least 1000 cells per sgRNA on average per replicate (>3E5 infected cells). The screens were run for around 14 doubling times and kept at >1000 cell coverage during each passage. Cells were sampled for genomic DNA extraction at different time points, as indicated in the screen timelines. DMSO or MEK inhibitors were added at indicated time points. Selumetinib was purchased from LC Laboratories. Trametinib, binimetinib and cobimetinib were purchased from MedChemExpress. Genomic DNA was extracted using GenElute™ Mammalian Genomic DNA Miniprep Kits (Merck, cat# G1N70). sgRNAs and MEK1 genomic locus were amplified using Phusion Hot Start II DNA Polymerase (Thermo Fisher) with primer pairs in Supplementary Table 9. The amplicons were gel purified using Wizard® SV Gel and PCR Clean-Up System (Promega, cat# A9282). NGS libraries were prepared by adaptor ligation and sequenced with 5 million 150 bp pair-end reads using Illumina NovaSeq (Eurofins Genomics). The exact cell coverages (average infected cell number per sgRNA) and timelines for each screen are as indicated in the screen protocol (see figures).

### Tiling screen data processing and analysis

MAGeCK (version 0.5.9.5) computational pipelines^54^ was used to extract read counts of each sgRNA from the FASTQ NGS raw files. Reads per million (RPM) was calculated by the following: RPM = (sgRNA counts/total counts in the sample) × 1 million. Log2 fold changes (LFCs) of the (RPM + 1) between two time points were calculated and provided as Supplementary Data 8. ROC analysis was generated by ROCit (version 2.1.1). Boxplots and density plots were generated by ggplot2 (version 3.3.5). Significance in boxplots was analysed by ggsignif (version 0.6.3) using unpaired two-tailed Wilcoxon test with 95% confidence interval. For the drop-out screen, sgRNAs with read counts below 300 in the A375 parental line day 0 sample were excluded for CRISPRO and structural analysis, to exclude potential outliers caused by low reads in NGS sequencing. CRISPRO (version 1.0.2) computational method^27^ was used to assign sgRNA LFC values to the MEK1 protein primary sequence and to generate the sgRNA bins. sgRNA on-target activity was predicted by CRISPRon deep learning model^30^. R (version 4.0.3) and RStudio (version 1.3.1093) were used to run R scripts.

### Structural analysis

NGL viewer (version 2.0.0)^55^ and PyMOL (The PyMOL Molecular Graphics System, Version 2.4.1 Schrödinger, LLC) was used to visualise the identified regions, highlight key residues, and superimpose protein 3D structures. Fpocket (version 4.0)^32^ was used to predict and score pockets in MEK1 structure (PDB 3EQI). AlphaFold (version 2.2)^37^ was used to generate homology models of the MEK1 variants. The homology models with the highest confidence were selected for structural analysis and are provided as Supplementary Data 9–14. For methods of molecular dynamics (MD) simulation, see Supplementary Note.

### Single locus editing by sgR234/L235

At day 0, Alt-R® CRISPR-Cas9 synthetic sgRNA targeting MEK1 R234 (sgR234/L235) (IDT) was transduced to A375-Cas9 cells in 6-well plates using Lipofectamine 2000 (Thermo Fisher) in two replicates. Cells were sampled at day 1 and day 9 for genomic DNA extraction. Amplicons around the sgRNA targeting site were obtained by PCR using Phusion Hot Start II DNA Polymerase (Thermo Fisher) with primer pair MEK1-LoF-F2/MEK1-LoF-R2 (Supplementary Table 9). Amplicons were gel purified and sent to Eurofins Genomics for NGS as described above. CRISPResso (version 2.2.11) computational method^56^ and Microsoft Excel (version 2320 Build 16.0.16130.20684) were used to align and process NGS sequencing data at each MEK1 locus. For all synthetic sgRNA sequences, see Supplementary Table 6. For NGS data processing and LFC value calculation, see Supplementary Data 5. The frameshift and in-frame INDEL mutations were classified to compare their depletions in group. And the LFC of each frameshift and in-frame mutations at day 2, day 5, and day 9 were calculated towards day 1 in each replicate. Statistical analysis was run by using R (version 4.2.2). Plotting and visualisation was done by using R packages ggplot2 (version 3.4.3). The analysed INDEL mutations, LFC values and mutation classes are listed in Supplementary Data 6.

### HA-tagged MEK1 plasmids cloning

MEK1 CDSs (wild-type, R234_L235, Q236_G237 and R234_G237 variants) were synthesised as gBlocks (IDT) and cloned into pcDNA™3.1 (+) Mammalian Expression Vector (Thermo Fisher) using *Not*I and *Bam*HI restriction enzymes (NEB) and T4 ligase from New England Biolabs (NEB). DNA sequence coding HA peptide was inserted at the N-terminus of MEK1 (after the ATG codon) using PCR, *Bam*HI/*Eco*RI restriction digestion and ligation. The constructs were validated by Sanger sequencing (Source BioScience). Plasmids are available upon request.

### Co-immunoprecipitation to detect the MEK1-BRAF interaction

Plasmids were transfected into HEK293 cells using Lipofectamine 2000 (Thermo Fisher) for 24 h. Transfected cells were lysed in 300 µL cell lysis buffer (0.5% NP40, 20 mM Tris pH7.5, 100 mM NaCl, 10% glycerol, 1 mM EDTA, pH 7.8) plus cOmplete™ protease and PhosSTOP™ phosphatase inhibitors (Merck). 1.5 mg of cell lysate was incubated with 50 µl Pierce™ Anti-HA Magnetic Beads (Thermo Fisher) at 4 °C rotating overnight. The next day, the beads were washed three times and resuspended in 30 µL cell lysis buffer. 60 µL of NuPAGE™ LDS Sample Buffer (Invitrogen) with 0.2 M DTT was added to the beads and boiled at 100 °C for 10 min. Following beads removal, supernatant was collected for western blotting.

### Drug treatment to cells

Cells were treated with different concentrations of MEK or BRAF inhibitors in 6-well plates for 2 h. They were then washed with 1 mL ice-cold PBS and lysed in the plates at 4 °C with 250 µL MSD Tris Lysis Buffer (Meso Scale Discovery) plus cOmplete™ protease and PhosSTOP™ phosphatase inhibitors (Merck). Cell lysate was collected by centrifuging at 14,000 rpm at 4 °C. Whole protein concentration in the lysate was measured by Pierce™ BCA Protein Assay Kit (Thermo Fisher), normalised and treated with LDS Sample Buffer for western blotting.

### Western blotting

Cell lysate or co-immunoprecipitation samples were separated by SDS-PAGE and transferred to nitrocellulose membranes. The primary antibodies are listed in Supplementary Table 8. Specific binding was detected using secondary antibodies and Odyssey Infra-red Imaging System (LI-COR, Cambridge, UK). All western blots were repeated at least two times and a representative blot was shown in the figures. Image Studio (Ver 5.2) was used for densitometry analysis.

### CRISPR/Cas9-mediated knock-in of MEK1 mutations

For CRISPR knock-in in A375-Cas9 line, cells were transduced with Alt-R® CRISPR-Cas9 synthetic sgRNAs (IDT) and Alt-R™ HDR Donor Oligo (IDT) using Lipofectamine 2000 (Thermo Fisher) for 48–72 h, with or without 0.5 µM Alt-R® HDR enhancer V2 (IDT). For HT-29 parental line, cells were electroporated with ribonucleoprotein (RNP) formed by mixing Alt-R® S.p. HiFi Cas9 Nuclease V3 (IDT) and Alt-R® CRISPR-Cas9 synthetic sgRNAs (IDT), together with Alt-R™ HDR Donor Oligo (IDT). 4D-NucleofectorTM X System (Lonza) was used for nucleofection with SF Cell Line 4D-Nucleofector™ X Kit S (Lonza) using the recommended protocol (Lonza Cell & Transfection database). Sequences of sgRNAs and HDR oligos used were listed in Supplementary Table 6. After nucleofection, cells were transferred to 6-well plates and incubated for 48–72 h. 0.1 µM binimetinib or 5 nM trametinib was added to the A375 or HT-29 CRISPR knock-in cell pools to maintain cells with MEK1 mutations. Single cell clones were isolated either by BD FACSMelody™ Cell Sorter (BD Biosciences) or dilutional cloning (5 cells/mL).

### Droplet digital PCR (ddPCR) genome edit detection assays

CRISPR/Cas9-mediated homology directed repair (HDR) was detected by droplet digital PCR (ddPCR). A duplicate of the 96-well plates with genome-edited single cells obtained as described above was generated. Cells in the duplicate plates were lysed using 100 µL QuickExtract™ DNA Extraction Solution (Lucigen) and transferred to 96-well PCR plates. Cell lysate containing 100 ng of genomic DNA was mixed with 1.1 µL HDR Genome Edit Detection Assay probe (20×) (MEK1_K59del, I111del, I141_C142delinsEI, G202_E203delinsEL or A372del probes) and 1.1 µL predesigned Reference Genome Edit Detection Assay probe (EIF2C1) (20 ×) (Bio-Rad) in 11 µL ddPCR Supermix for Probes no dUTP (2×) (Bio-Rad) with 0.11 µL FastDigest HindIII restriction enzyme (Thermo Fisher) to a final volume of 22 µL. The reaction pools were then processed by an Automated Droplet Generator (Bio-Rad). PCR reaction was immediately run on a C1000 Touch Thermo Cycler (Bio-Rad) with the following programme: 95 °C 10 min, 40 cycles of 94 °C 30 s then 55 °C 2 min 30 s, 98 °C 10 min, 4 °C storage. Plates were read by a QX200 Droplet Reader (Bio-Rad). The data was analysed using QuantaSoft Software (Bio-Rad). The knock-in loci of the positive single cell clones were PCR amplified using primer pairs indicated in Supplementary Table 9 and sequenced using Amplicon-EZ next generation sequencing service (GENEWIZ). Genotyping was done by analysing sequencing data using CRISPResso computational method. All CRISPResso analysis was performed by Eurofins Genomics.

### Cell viability assays

A375 or HT-29 cells were seeded into 96-well plates at density of 2 k or 4 k cells per well the day before drug treatment. DMSO dissolved drugs were added to cells in serial dilution with final 0.1% DMSO concentration. A375 or HT-29 cells were incubated with drugs for 96 h or 120 h, respectively. A375 cell viability (metabolism) was measured using alamarBlue® reagent (Bio-Rad). 20 µL alamarBlue® was added to each well and incubation 6 h at 37 °C in incubator. Fluorescence in each well was read at 535 nm/590 nm on a Gemini XPS Microplate Reader (Molecular Devices). Value of each well, minus mean no-cell value, was expressed as a percentage of the mean DMSO control value. Sigmoidal dose-response (variable slop) curve fit and IC_50_ value determination were performed on GraphPad Prism (version 9.0.2) (GraphPad Software). All dose response curves were repeated at least three times and a representative one was shown in figures.

### Live cell imaging by IncuCyte

Live cell images were taken and analysed by Incucyte® SX5 (Essen BioScience). IncuCyte® Live-Cell Analysis Systems (with software 2022 A, version 20221.1.0.0) was used to measure and calculate cell confluency from non-overlapping phase contrast images of each well. Confluency of HT-29 cell lines was followed for 5 days with addition of DMSO dissolved drugs in serial dilution (with a final concentration of 0.1% DMSO). The measurements of cell confluency were repeated at least two times and a representative one was shown in figures. HT-29 cell proliferation was indicated as percentage of confluency of treatment normalised to DMSO at day 5. Cell nuclei were stained with IncuCyte® NucLight Rapid NIR Reagent (Essen BioScience) for 2 h before imaging and were counted by Incucyte 2022 A software (version 20221.1.0.0). HT-29 cell metabolism was calculated as alamarBlue® measurements normalised to cell nuclei counts at day 5. The measurements of metabolism per cell were repeated at least four times and a representative one was shown in figures.

### Statistics

GraphPad Prism (version 9.0.2) (GraphPad Software) was used for statistical analysis. Unpaired two-tailed t-tests were used for significance analysis of dose response curve IC_50_s and normalised cell metabolism, using a confidence level of 95%. Significant values as well as number of replicates are noted for each experiment in the respective figure legends. All other statistical methods are described in their respective method sections.

### Reporting summary

Further information on research design is available in Nature Portfolio Reporting Summary linked to this article.

## Supplementary information

Supplementary Information

Description of Additional Supplementary File

Supplementary Data 1-18

Reporting Summary

## Data Availability

The sgRNA library, CRISPR tiling screen data, CRISPRO outputs, sgR234/L235 single locus editing data, AlphaFold homology models, oligonucleotide sequences, antibodies used in western blot, detailed methods for MD simulation and mutation information from GENIE dataset are provided as Supplementary Information. The following publicly available datasets were used: PDB accession codes 3EQI, 6V2W, 1S9J, 7JUZ, 7M0U, 7M0Y, 7M0Z, 4ANB; ClinVar (https://www.ncbi.nlm.nih.gov/clinvar/) and PDBePISA (https://www.ebi.ac.uk/pdbe/pisa/). Source data for graphs is provided as Supplementary Data 16–18. Uncropped and unedited blot images are provided as Supplementary Figs. 9–12. All other data are available on reasonable request.

## Code availability

Codes for MAGeCK (https://sourceforge.net/p/mageck/wiki/Home/), CRISPRO, NGLviewer (https://github.com/nglviewer/ngl), CRISPResso (https://github.com/pinellolab/CRISPResso2) and Fpocket (https://github.com/Discngine/fpocket) computational methods are publicly available. Basic R scripts used for RPM calculation, log2 fold change calculation and plotting are available upon request.
