# Supplementary material for: The identification of functional regions of MEK1 using CRISPR tiling screens
Source: Commun Biol. 2025 Apr 24;8:656. doi: 10.1038/s42003-025-07966-4 (PMC12022096; doi:10.1038/s42003-025-07966-4)
Supplement: Supplementary file 1 — Supplementary Information [file 42003_2025_7966_MOESM1_ESM.pdf]

Contents of Supplementary Information

Supplementary Figures

Supplementary Figure 1. CRISPRO sgRNA bins for all tiling screens and MEK1 structures colour-coded by the corrected drop-out screen LFC values.

Supplementary Figure 2. Assessment of functional relevance of Helix EF region targeted by sgR234/L235.

Supplementary Figure 3. Tiling screen sgRNA LFC values mapped to MEK1 primary sequences.

Supplementary Figure 4. Genotyping and verification of selected dominant MEK1 drug resistant mutations.

Supplementary Figure 5. Genotyping MEK1 mutations in the drug-resistant A375 cell pools.

Supplementary Figure 6. Verification of the drug-resistant mutations in HT29 cell line.

Supplementary Figure 7. Comparison of conformational changes and differences to an active structure between the MEK1 wild type and A372del variant ensembles.

Supplementary Figure 8. Comparison of the representative conformations of the simulated ensembles between their di-phosphorylated and non-phosphorylated forms.

Supplementary Tables

Supplementary Table 1. Summary of the MEK1 sgRNA library content.

Supplementary Table 2. Most depleted MEK1 mutants introduced by sgR234/L235.

Supplementary Table 3. Least depleted MEK1 mutants introduced by sgR234/L235.

Supplementary Table 4. Mutations in tumours around the two novel drug resistance regions.

Supplementary Table 5. Classification of mutations around the two novel drug resistance regions.

Supplementary Table 6. sgRNA and HDR oligo sequences.

Supplementary Table 7. Summary of MEK1 drug resistance regions and mutants.

Supplementary Table 8. List of antibodies used in this paper.

Supplementary Table 9. List of primers used in this paper.

Supplementary Note

Methods for Molecular Dynamics simulation.

Supplementary Figures

a

	1	2	3	4	5	6	7	8	9	10	11	12	13	14	15	16	17
**Drop-out**	-5.02	-4.35	-3.68	-3.01	-2.34	-1.67	-1.00	-0.33	0.33	1.00	1.67	2.34	3.01	3.68	4.35	5.02	
**Selumetinib**	-4.77	-4.13	-3.50	-2.86	-2.23	-1.59	-0.95	-0.32	0.32	0.95	1.59	2.23	2.86	3.50	4.13	4.77	
**Trametinib**	-3.34	-2.89	-2.45	-2.00	-1.56	-1.11	-0.67	-0.22	0.22	0.67	1.11	1.56	2.00	2.45	2.89	3.34	
**Cobimetinib**	-3.67	-3.18	-2.69	-2.20	-1.71	-1.22	-0.73	-0.24	0.24	0.73	1.22	1.71	2.20	2.69	3.18	3.67	
**Binimetinib**	-5.00	-4.33	-3.67	-3.00	-2.33	-1.67	-1.00	-0.33	0.33	1.00	1.67	2.33	3.00	3.67	4.33	5.00	
**crispron**	-9.06	-7.85	-6.64	-5.44	-4.23	-3.02	-1.81	-0.60	0.60	1.81	3.02	4.23	5.44	6.64	7.85	9.06	

b

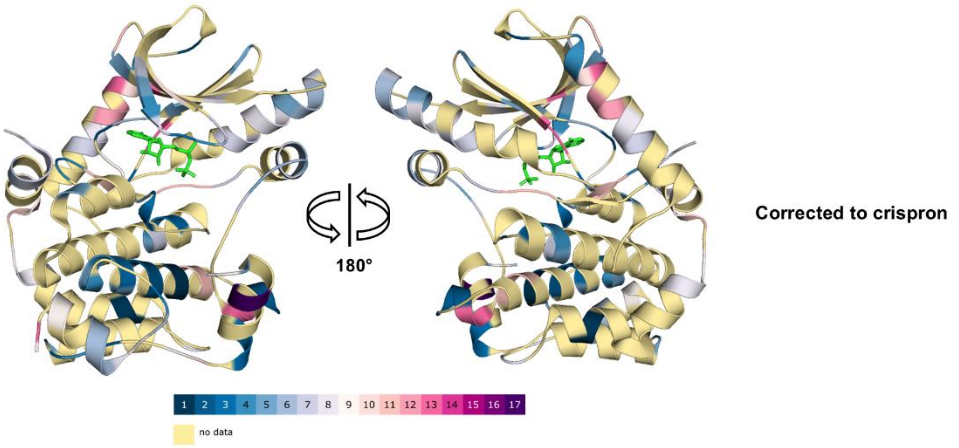

Supplementary Fig. 1. CRISPRO sgRNA bins for all tiling screens and MEK1 structures colour-coded by the corrected drop-out screen LFC values. a. Cut-off LFC values are listed below the colour codes of the 17 sgRNA bins. Bins of each screen (drop-out, selumetinib, trametinib, cobimetinib and binimetinib screen), and bins of drop-out screen corrected to CRISPRon are shown. **b.** MEK1 structures colour-coded by the corrected sgRNA LFC values. MEK1 residues are colour-coded by sgRNA bins in a heatmap style. Colour codes of the sgRNA bins are indicated below the structures. Molecule in green: ADP. PDB code: 3EQI.

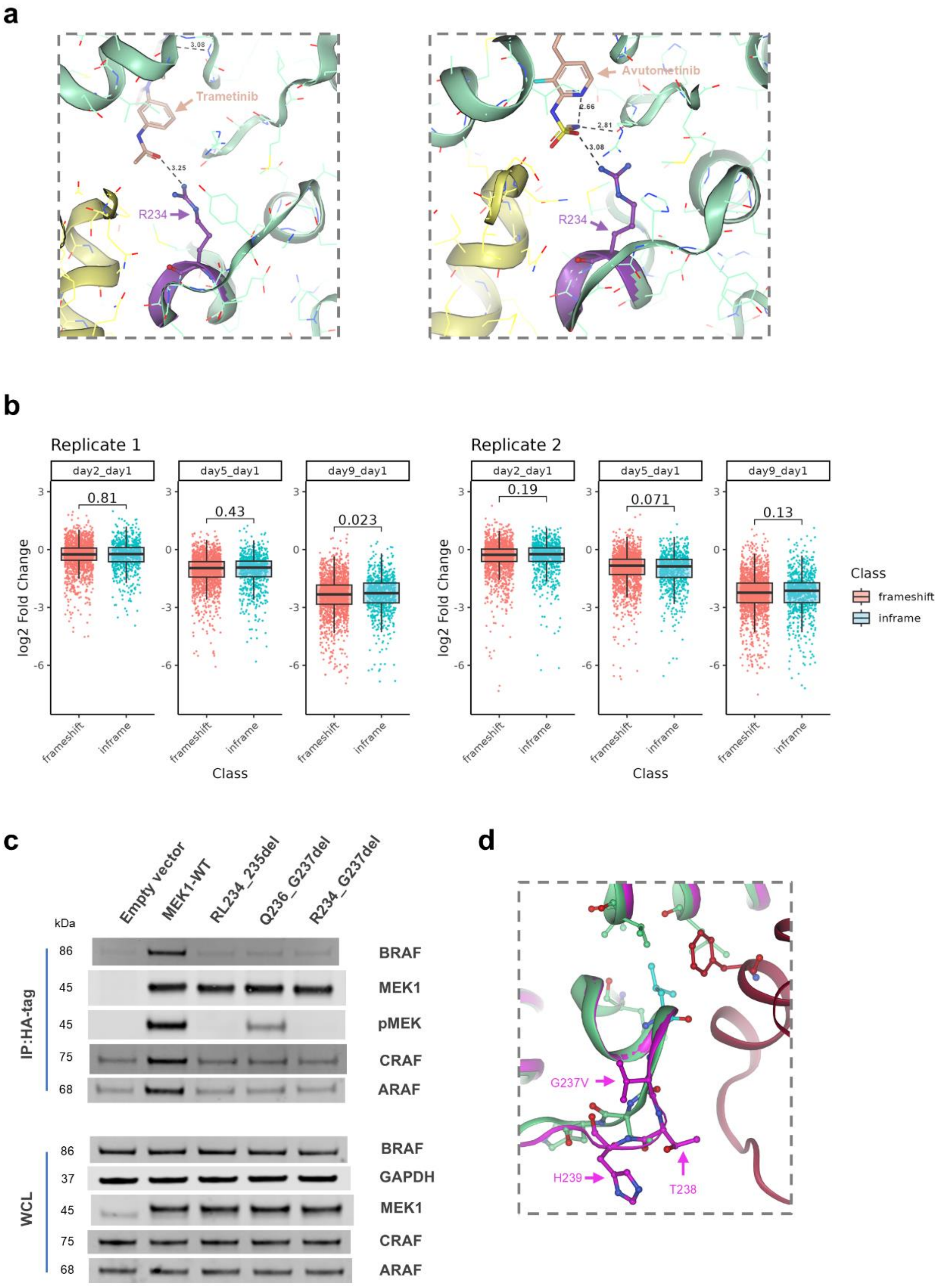

Supplementary Fig. 2. Assessment of functional relevance of Helix EF region targeted by sgR234/L235. a. Structural visualization of MEK1 inhibitors interacting with R234. Polar interactions are indicated by black dashed lines. PDB ID: 7JUR (left) and 7M0Z (right). **b.** Boxplots showing comparison of depletion between frameshift and in-frame INDEL mutations generated by sgR234/L235**.** LFC values of three time points of two replicates were compared. p-values are labelled as numbers on top of the boxes. **c.** Co-immunoprecipitation of small

deletion mutants. IP: immunoprecipitation. WCL: whole cell lysate. **d.** Overlay of MEK1 G237V homology model with. wild-type structure (PDB: 6V2W). MEK1 G237V homology model is in magenta, MEK1 wild-type in green and BRAF in dark red.

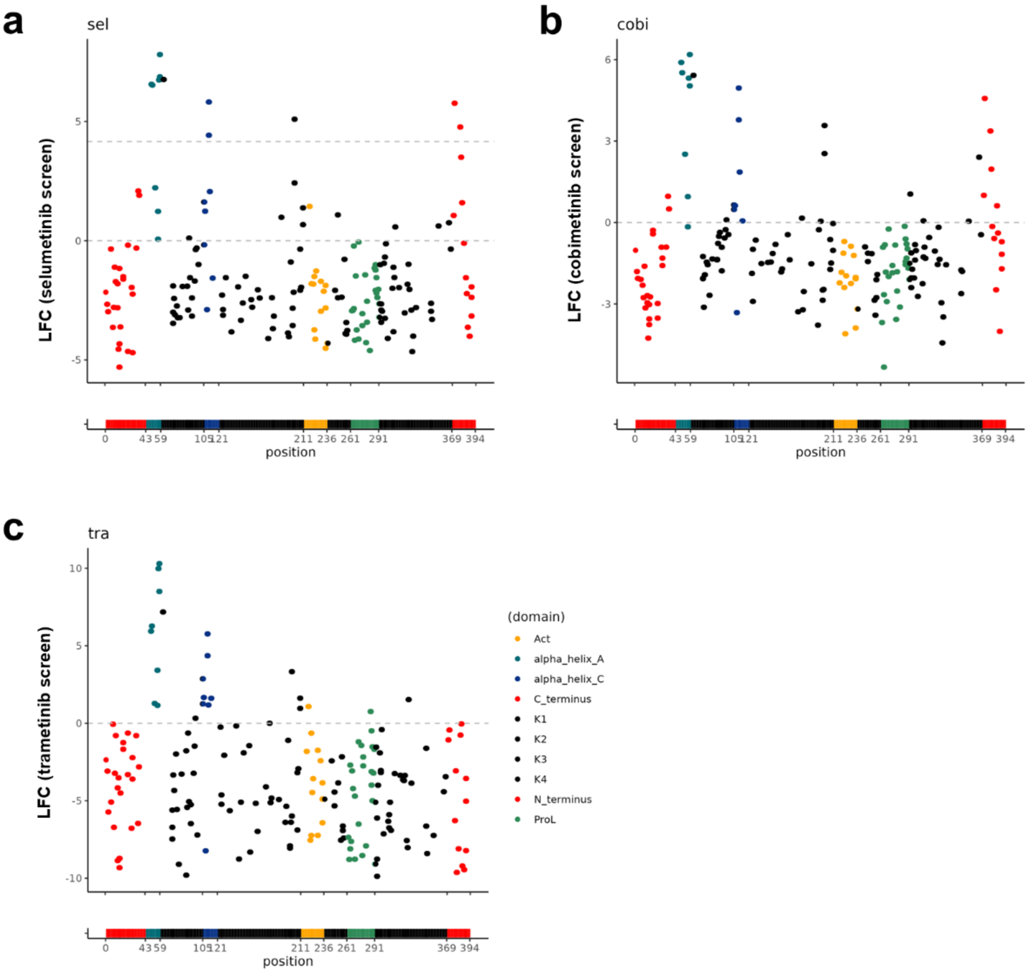

Supplementary Fig. 3. Tiling screen sgRNA LFC values mapped to MEK1 primary sequences. LFC values of selumetinib (**a**), cobimetinib (**b**) and trametinib (**c**) screens were plotted. Dots are coloured according to MEK1 domains. A-loop: activation loop, K1-K4: core kinase domain 1-4, P-rich loop: proline-rich loop. Data points represent mean of two replicates.

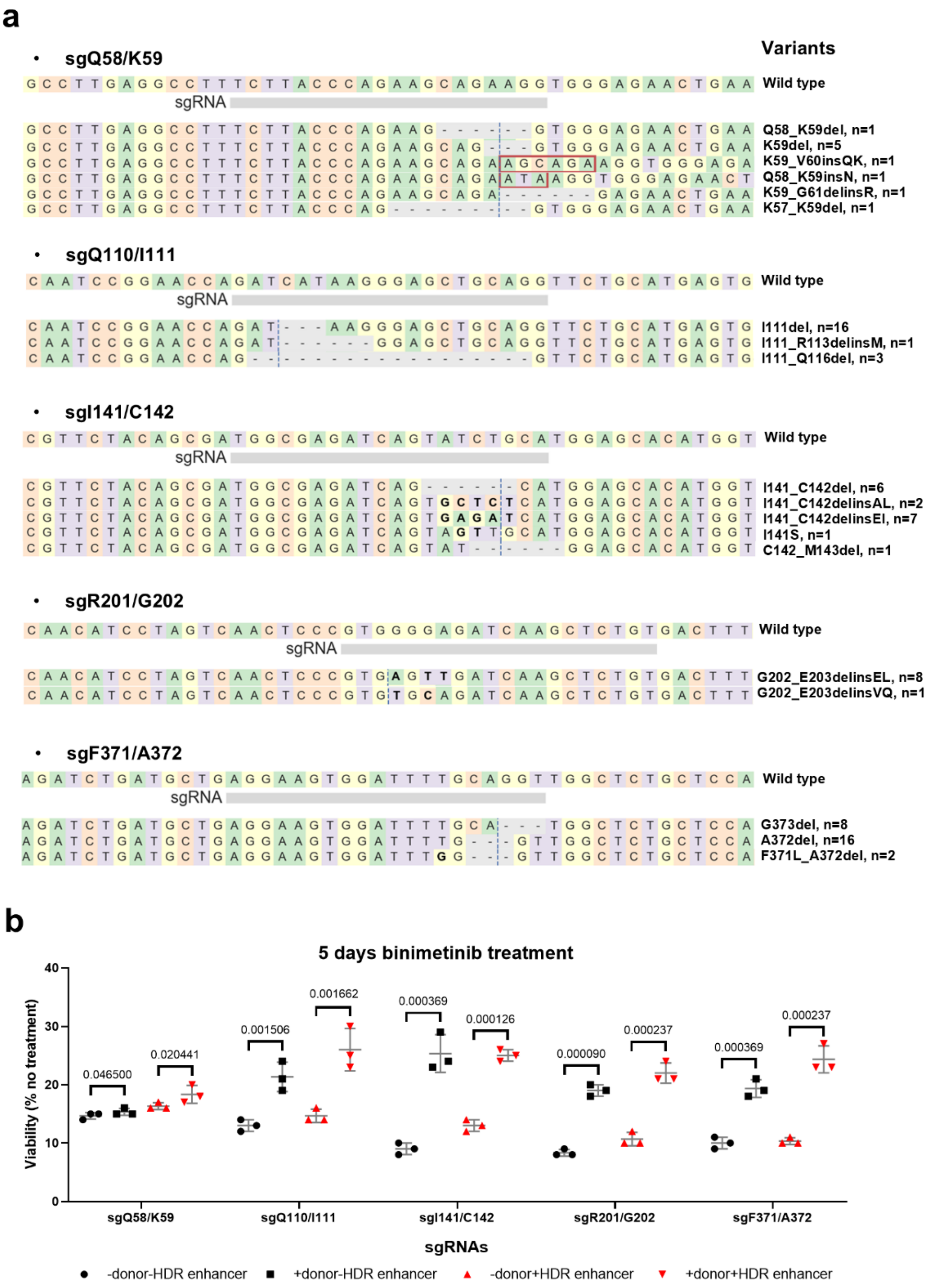

Supplementary Fig. 4. Genotyping and verification of selected dominant MEK1 drug resistant mutations. a. Genotyping of MEK1 mutations in the drug resistance single cell clones of the A375 cell line. All genotyped MEK1 in-frame mutations were aligned to the wild-type genomic DNA sequence at each site. The protein variants resulting from the mutations are indicated as "Variants" following each nucleotide sequence. n, the number of times a variant allele was identified in all genotyped drug resistance clones. Alignments were created by CRISPResso computational method. sgRNA binding sites were indicated by grey bars below the wild-type sequences. Cas9 cutting sites are indicated by dashed blue lines.

Nucleotide insertions are indicated in red boxes. Nucleotide substitutions are indicated in **bold** font. Nucleotide deletions are indicated as "–". **b.** Viability assay comparing sgRNA alone and sgRNA + HDR donor on generating binimetinib resistance. +/- donor: with or without HDR donor template, +/- HDR enhancer: with or without Alt-R™ HDR enhancer. q-values are indicated above the columns, calculate by unpaired multiple t tests, using two-stage step-up method of Benjamini, Krieger and Yekutieli. Error bars represent standard deviations of 3 replicates.

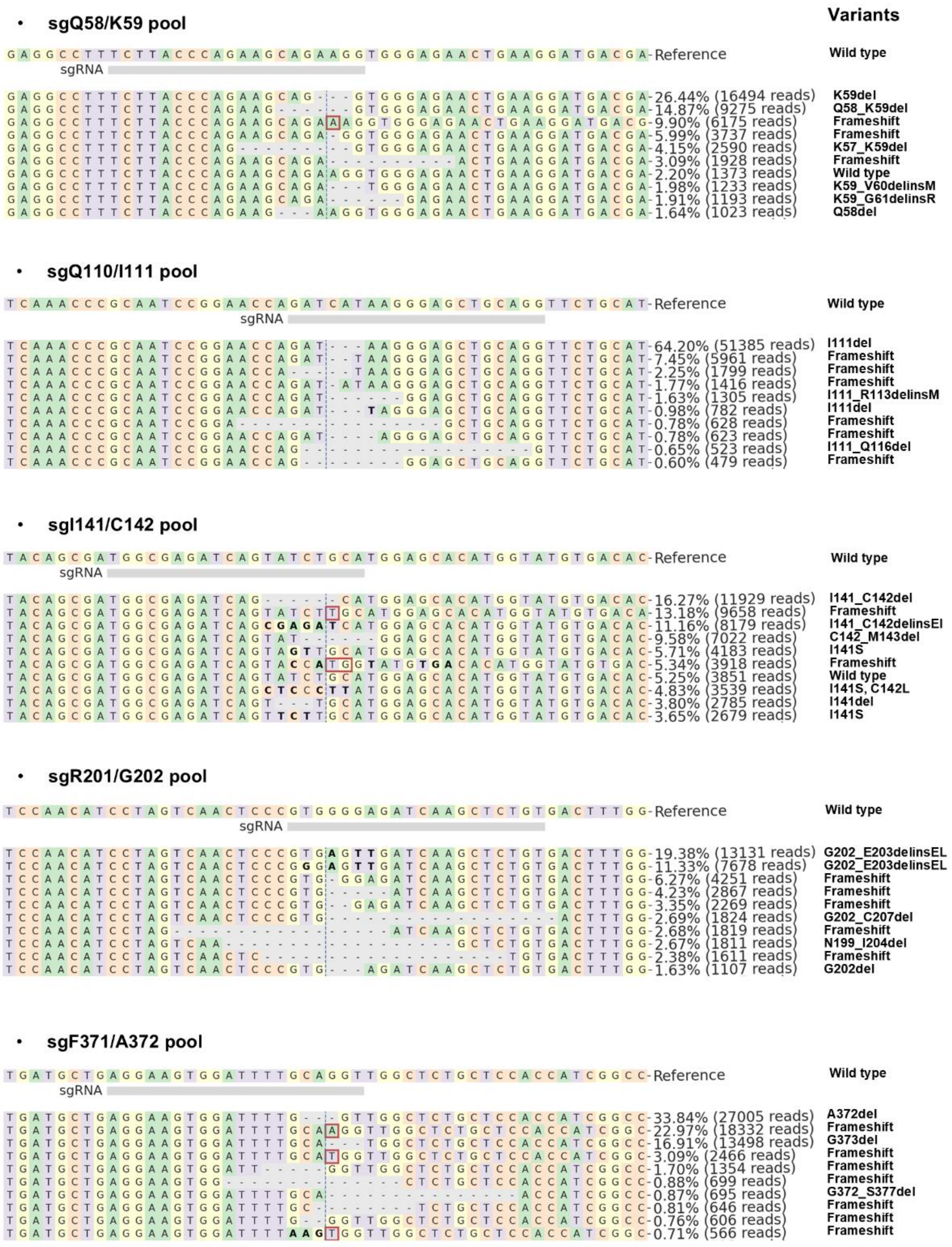

Supplementary Fig. 5. Genotyping MEK1 mutations in the drug-resistant A375 cell pools. The genotyped MEK1 mutations at each site were aligned to the wild-type DNA sequences and the top 10 abundant mutations at each site were listed. The frequency of each mutant allele in the cell pool was indicated as percentage followed by NGS read counts. MEK1 variants are indicated following the nucleotide sequences (frameshift mutations indicated as “Frameshift”). Alignment was created by CRISPResso computational method. sgRNA binding sites were indicated by grey bars below the wild-type sequences. Cas9 cutting sites are indicated by dashed blue lines. Nucleotide insertions are indicated in red boxes. Nucleotide substitutions are indicated in **bold** font. Nucleotide deletions are indicated as “–”.

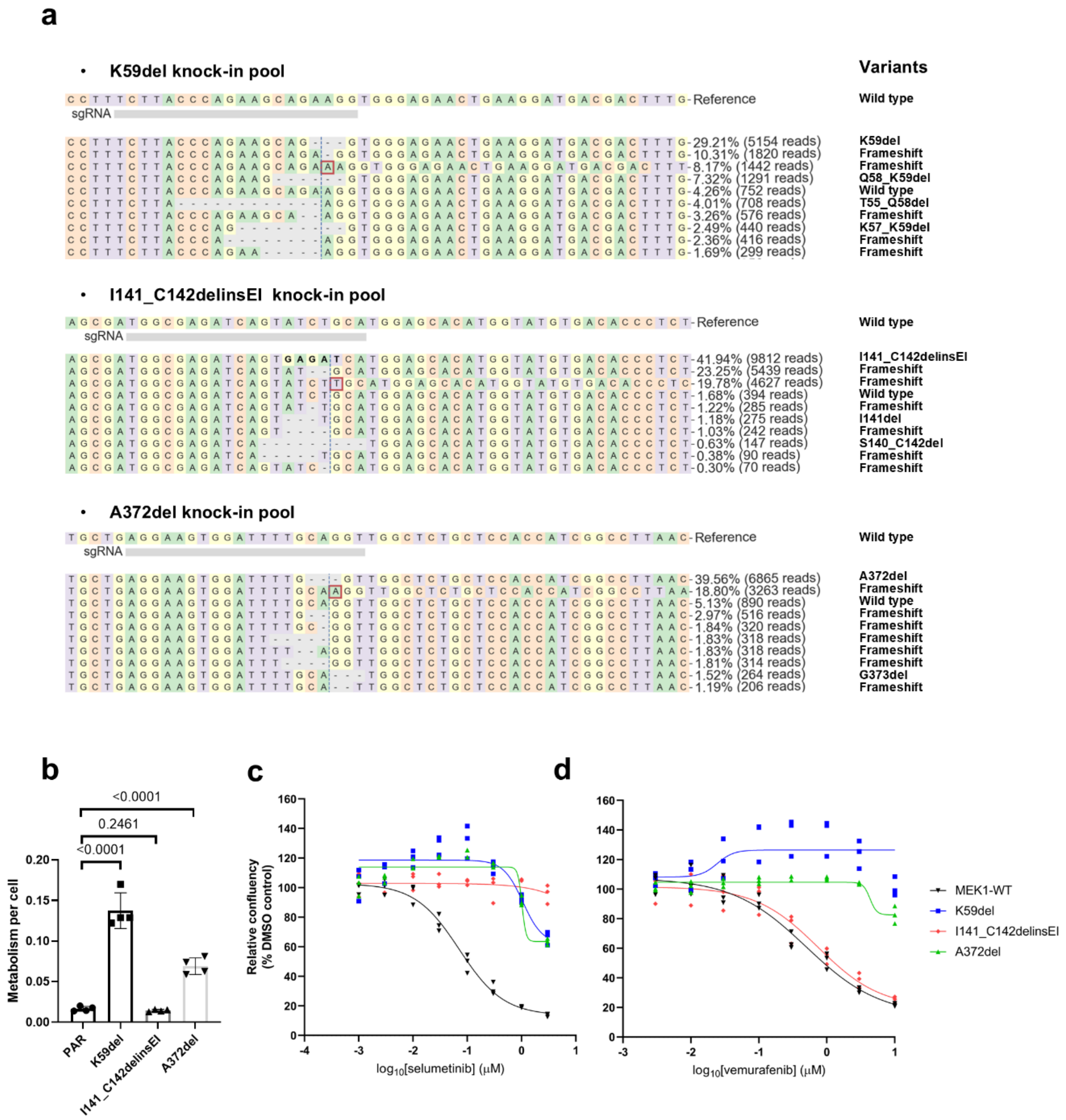

Supplementary Fig. 6. Verification of the drug-resistant mutations in HT29 cell line. a. Genotyping of MEK1 mutations in drug selected CRISPR knock-in cell pools of the HT-29 cell line. The genotyped MEK1 mutations at each site were aligned to the wild-type DNA sequences and the top 10 abundant mutations at each site were listed. The frequency of each mutant allele in the cell pool was indicated as percentage followed by NGS read counts. MEK1 variants are indicated following the nucleotide sequences (frameshift mutations indicated as "Frameshift"). Alignment was created by CRISPResso computational method. sgRNA binding sites were indicated by grey bars below the wild-type sequences. Cas9 cutting sites are indicated by dashed blue lines. Nucleotide insertions are indicated in red boxes. Nucleotide substitutions are indicated in **bold** font. Nucleotide deletions are indicated as "–". **b.** Comparison of the normalised cell basal metabolism between the HT-29 parental line (PAR) and gene edited cell lines. Basal metabolism levels were normalised to the counts of stained cell nuclei (as metabolism per cell). p-values are shown above the columns. Error bars

represent SD. N=4. **c-d.** Dose response curves of HT-29 cell line proliferation in response to selumetinib (**c**) or vemurafenib (**d**). Cell confluency is shown as percentage to the DMSO control. Data points with three replicates are shown.

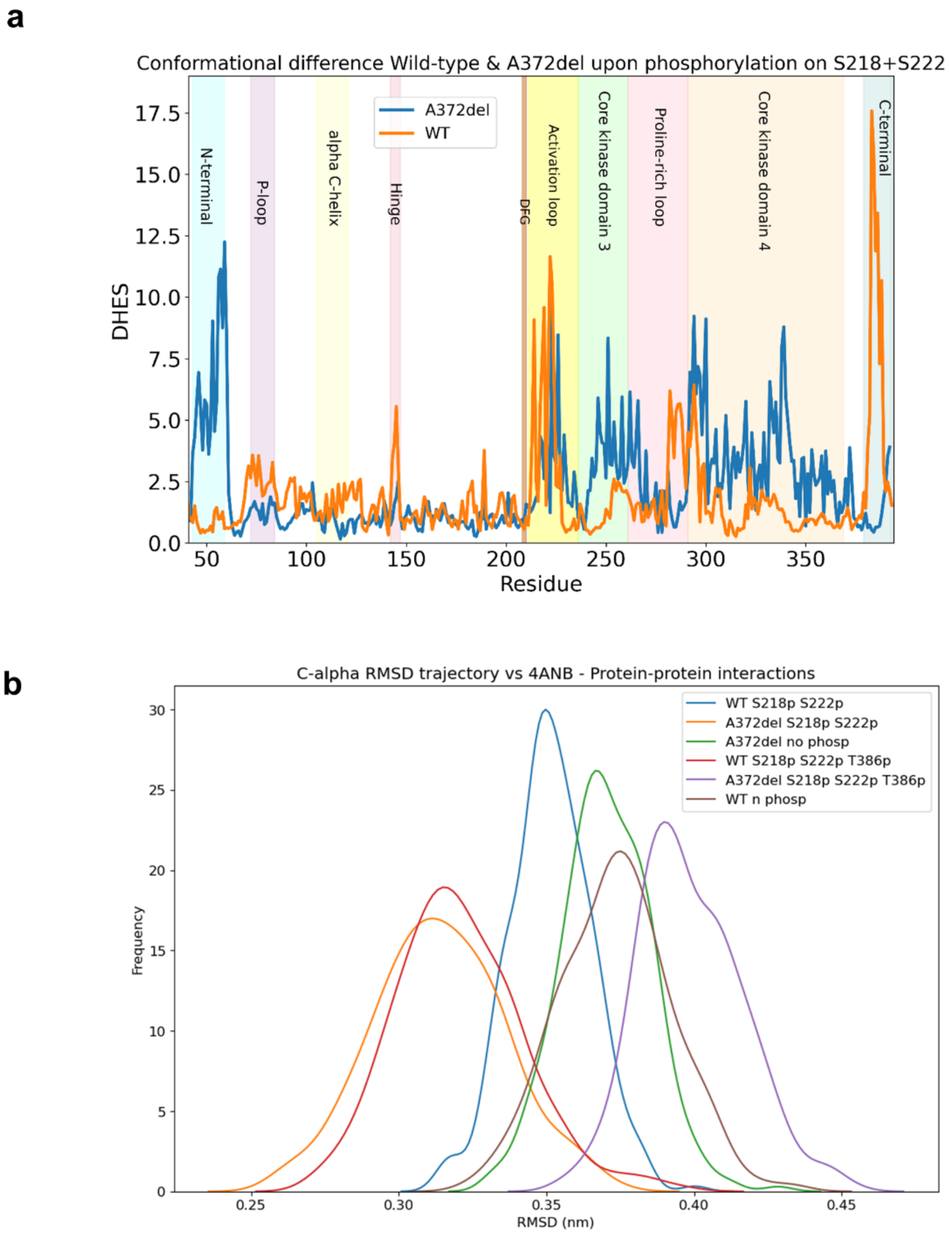

Supplementary Fig. 7. Comparison of conformational changes and differences to an active structure between the MEK1 wild type and A372del variant ensembles. A. The pairwise D_{HES} between the non-phosphorylated and the di-phosphorylated forms of the ensembles. The MEK1 domains, on which the residues lie, are indicated. **B.** Conformational difference between the simulated ensembles and the active MEK1 crystal structure. C-alpha RMSD distributions were obtained by comparing the trajectories of each simulated ensemble with the S218D/S222D double mutant MEK1 crystal structure (PDB code: 4ANB). MEK1 residues involved in the MEK1-BRAF PPI sites were compared, including 309-312, 314-315, 318-319, 78, 102-106, 192, 213, 219-228, 230, 307 and 234-238.

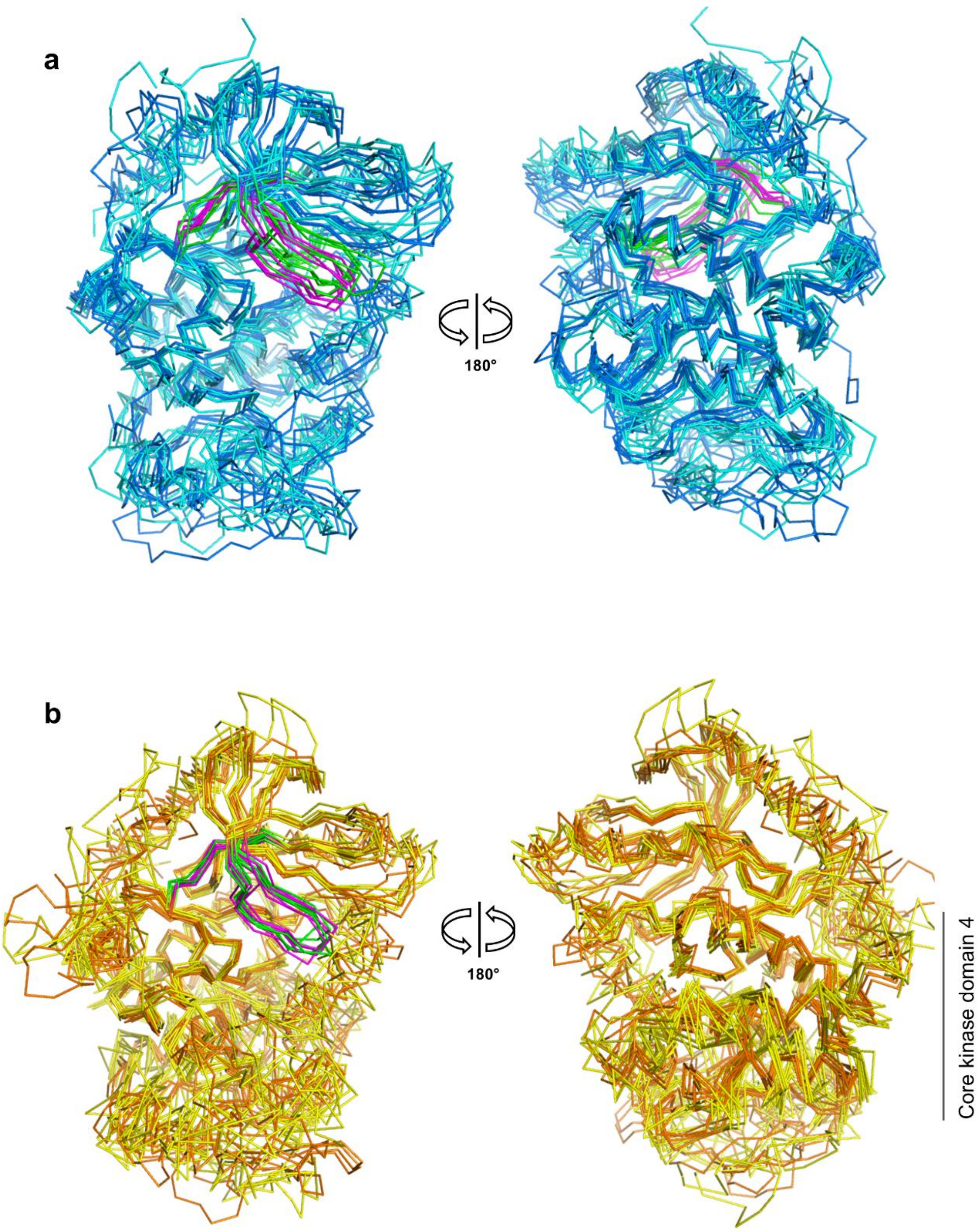

Supplementary Fig. 8. Comparison of the representative conformations of the simulated ensembles between their di-phosphorylated and non-phosphorylated forms. **A.** Representative conformations of the inactive, non-phosphorylated forms of MEK1 wild type are coloured in cyan, and the di-phosphorylated forms in dark blue. The P-loop and hinge motif of the non-phosphorylated forms are coloured in green, and the di-phosphorylated forms in magenta. **B.** Representative conformations of the inactive, non-phosphorylated forms of A372del variant are coloured in yellow, and the di-phosphorylated forms in orange. The P-loop and hinge motif of the non-phosphorylated forms are coloured in green, and the di-phosphorylated forms in magenta. Core kinase domain 4 is indicated.

Supplementary Tables

Supplementary Table 1. Summary of the MEK1 sgRNA library content.

sgRNA category	Target gene	A375 dependency	sgRNA number
CDS-targeting	MAP2K1	-1.25	187
Non-coding		NA	38
Non-targeting	EGFP	NA	29
Positive controls	VCP	-2.41	6
	PCNA	-2.44	6
	EEF2	-2.31	6
Negative controls	EEF2K	0.011	6
	DCC	-0.019	6
	BACE2	0.065	6
	LAMA1	0.095	6
	NRAS	-0.093	4
Total			300

Supplementary Table 2. Most depleted MEK1 mutants introduced by sgR234/L235.

Replicate 1			Replicate 2	
Mutant	LFC	Rank	Mutant	LFC
**R234G,L235del**	-2.71	1	**R234G,L235del**	-2.55
**L235_Q236del**	-2.67	2	**R234_L235del**	-2.46
**E233D,R234_L235del**	-2.64	3	**Q236_T238del**	-2.42
Q236_G237del	-2.46	4	R234_L235insL	-2.39
**R234_L235del**	-2.46	5	**L235del**	-2.38
**L235del**	-2.45	6	Q236del	-2.36
R234_L235delinsS	-2.43	7	Q236_G237del,T238A	-2.35
**Q236_T238del**	-2.43	8	**L235_Q236del**	-2.35
**E233_L235del**	-2.40	9	**E233D,R234_L235del**	-2.30
Q236_G237del	-2.35	10	**E233_L235del**	-2.28
Frameshift D10	-2.36	POS	*Frameshift D10*	-2.26
Frameshift D1	-2.58	POS	*Frameshift D1*	-2.57
Frameshift D11	-2.52	POS	*Frameshift D11*	-2.35

*Note: consensus mutants between replicates are highlighted in **bold**.

Supplementary Table 3. Least depleted MEK1 mutants introduced by sgR234/L235.

Replicate 1			Replicate 2	
Mutant (SNVs)	LFC	Rank	Mutant (SNVs)	LFC
**Wild type**	0.68	1	**Wild type**	0.66
**G237V**	0.31	2	**G237V**	0.60
**G237W**	0.09	3	**Q236H**	0.15
Q236K	-0.15	4	**G237W**	0.11
**Q236H**	-0.16	5	**L235I**	-0.19
**L235= (CTC to CTA)**	-0.19	6	**L235= (CTC to CTA)**	-0.26
**L235= (CTC to CTG)**	-0.38	7	**L235= (CTC to CTG)**	-0.37
**L235I**	-0.39	8	**R234I**	-0.40
**R234I**	-0.52	9	**L235= (CTC to CTT)**	-1.15
**L235= (CTC to CTT)**	-1.08	10		

*Note: consensus mutants between replicates are highlighted in **bold**.

Supplementary Table 4. Mutations in tumours around the two novel drug resistance regions.

Start Position	End Position	Variant Classification	Reference Allele	Tumor Seq Allele1	Tumor Seq Allele2	MEK1 protein mutant
66782879	66782879	Missense Mutation	G	G	C	D370H
66782882	66782882	Missense Mutation	T	T	A	F371I
66782901	66782901	Missense Mutation	C	C	T	S377F
66782904	66782904	Missense Mutation	C	C	T	T378I
66782908	66782908	Missense Mutation	C	C	G	I379M
66782909	66782909	Missense Mutation	G	G	A	G380S
66782924	66782924	Missense Mutation	A	A	C	S385R
66729210	66729210	Missense Mutation	A	A	C	S140R
66729211	66729211	Missense Mutation	G	G	T	S140I

Supplementary Table 5. Classification of mutations around the two novel drug resistance regions.

Start Position	End Position	Variant Classification	Variants	Condition(s)	Significance*
66782886	66782886	Missense Mutation	A372V	RASopathy, not provided	VUS
66782901	66782901	Missense Mutation	S377F	Noonan syndrome and Noonan-related syndrome	VUS
66782906	66782906	Missense Mutation	I379V	RASopathy	VUS
66782909	66782909	Missense Mutation	G380S	Noonan syndrome and Noonan-related syndrome, RASopathy	VUS
66782915	66782915	Missense Mutation	N382H	Melanoma	P
66729213	66729213	Missense Mutation	I141V	RASopathy	VUS
66729219	66729219	Missense Mutation	M143V	Cardiofaciocutaneous syndrome 3	LP

*VUS, variant of uncertain significance; P, pathogenic variant; LP, likely pathogenic variant.

Supplementary Table 6. sgRNA and HDR oligo sequences for CRISPR knock-in of MEK1 mutations.

sgRNA sequence	Targeting site	HDR oligo sequence
TCTTACCCAGAAGCAGAAGG	Q58/K59	GCAGCGAAAGCGCCTTGAGGCCTTTCTTACCCAGAAGCAGGTGGGAGAACTGAAGGATGACGACTTTGAGAAGATCAGTG
CCTGCAGCTCCCTTATGATC	Q110/I111	TAATTCATCTGGAGATCAAACCCGCAATCCGGAACCAGATAAGGGAGCTGCAGGTTCTGCATGAGTGCAACTCTCCGTAC
TGGCGAGATCAGTATCTGCA	I141/C142	GGGCTTCTATGGTGCGTTCTACAGCGATGGCGAGATCAGTGAGATCATGGAGCACATGGTATGTGACACCCTCTCAGCCTCTGGA
ACAGAGCTTGATCTCCCCAC	R201/G202	GCAGATGTCAAGCCCTCCAACATCCTAGTCAACTCCCGTGAGTTGATCAAGCTCTGTGACTTTGGGGTCAGCGGGCAGCTCATC
AGGAAGTGGATTTTGCAGGT	F371/A372	GCTTTTATCAAGAGATCTGATGCTGAGGAAGTGGATTTTGGTTGGCTCTGCTCCACCATCGGCCTTAACCAGCCCAGCAC
TGCAGCCAGAAAGACTCCAG	R234/L235	-

Supplementary Table 7. Summary of MEK1 drug resistance regions and mutants.

Regions identified	Location	Mutation hotspots	Known as resistance	Resistance mechanism	MEK1 mutants studied
R49_E51, K57_K59, E62/L63	Helix A	Yes	Yes, clinical & literature	MEK1 Activating	K59del
Q110/I111	Helix C	No	Yes, clinical &literature	Pocket perturbation & MEK1 Activating	I111del
I141/G142	Allosteric pocket	No	No	Pocket perturbation	I141_C142delinsEI
R201/G202	β7-β8 loop	Yes	Yes, clinical & literature	MEK1 Activating	G202_E203delinsEL
F371/A372, S377/T378	C-terminus	No	No	MEK1 Activating	A372del

Supplementary Table 8. List of antibodies used in this paper.

Antibody	Brand & Cat#	Concentration used for WB
tMEK	CST 4694	1:1000
pMEK	CST 9154	1:1000
tERK	CST 4696	1:1000
pERK	CST 4370	1:2000
tRSK	R&D Systems MAB2056	1:1000
pRSK	CST 8753	1:1000
pCRAF	CST 9431	1:1000
GAPDH	CST 5174	1:3000
tMEK1	CST 2352	1:1000
pMEK1-T386	Abcam 254096	1:1000
tBRAF	CST 60099	1:500
HA-tag	CST 3724	1:2000
Cas9	Abcam 191468	1:500
tCRAF	CST 53745	1:500
tARAF	CST 4432	1:1000

Supplementary Table 9. List of primers used in this paper.

Primer label	Sequence	Used for
U6-F	GGACTATCATATGCTTACCGTA	PCR of sgRNAs
sgRNA-R	TTCAAGTTGATAACGGACTAGC	PCR of sgRNAs
MAP2K1-HA-R	TGTTCAGGAATTCTTCCAGC	MEK1 N-terminus HA-tagging
MAP2K1-HA-F	CTCGGATCCACTAGTCCAGTGTGGTGGATGTACCCAT ACGATGTTCCAGATTACGCTCCCAAGAAGAAGCCGAC	MEK1 N-terminus HA-tagging
MAP2K1-PCR-R	TCGAGCGGCCGCTTAGACGCCAGCAGCATG	MEK1 cloning into pcDNA3.1(+)
MAP2K1-PCR-F	GCTCGGATCCATGCCCAAGAAGAAGCCG	MEK1 cloning into pcDNA3.1(+)
MAP2K1-NGS-F1	TGGAACAGGACCAACTTG	PCR and NGS of region ~K59
MAP2K1-NGS-R1	AGACCTTGAACACCACAC	PCR and NGS of region ~K59
MAP2K1-NGS-F4	TATATCTTTCATCCCTTCCTCC	PCR and NGS of region ~I111
MAP2K1-NGS-R2	TCCATGCAGATACTGATCTC	PCR and NGS of region ~I111
MAP2K1-NGS-F16	CCAGATCATAAGGGAGCTG	PCR and NGS of region ~I141
MAP2K1-NGS-R6	CTCCCAGACCAAAGATTAGG	PCR and NGS of region ~I141
MAP2K1-NGS-F13	TGGAATGCTGATCCTTCTC	PCR and NGS of region ~R201
MAP2K1-NGS-R5	CAAGAAGCTCAAGCAATGG	PCR and NGS of region ~R201
MAP2K1-NGS-F10	CGTCCTCTCGTTTCCTTAC	PCR and NGS of region ~A372
MAP2K1-NGS-R4	TGAACAGAGACAGGCATG	PCR and NGS of region ~A372
MEK1-LoF-R2	AAACATCAGCTCCAGCTC	PCR and NGS of region ~R234
MEK1-LoF-F2	TGGAGCTCTTGAAACAGG	PCR and NGS of region ~R234
BGH-R	TAGAAGGCACAGTCGAG	Sequencing primer for pcDNA3.1(+)
M13F	TGTAAAACGACGGCCAGT	Sequencing primer for pcDNA3.1(+)

Supplementary Note

Methods for Molecular Dynamics Simulation

System setup for MD of wild-type and A372del mutants

In order to study the effects of the A372del mutation in MEK1, three protein states were simulated: an inactive state with no phosphorylation, an activated state phosphorylated in S218 and S222 and a triple phosphorylated state with phosphorylation on S218, S222 and T386. These three states were modelled for both wild-type and mutant, resulting in a total of six simulated systems.

The starting point for the simulations were AlphaFold2[1] structures of both A372del mutant and wild-type MEK1. It was necessary to use MEK1 homology models because x-ray structures lack the C-terminal region where the T386 phosphorylation site is located, along with other segments comprising the proline rich loop and part of the core kinase domain 3. The structures were truncated from the 41 to 393 residues.

ATP and Mg^{2+} were introduced in the active site by superposing the x-ray structure with the PDB code 3EQD[2]. The protonation states were determined at pH 7.0 using the PROPKA protonation estimator[3] built in Maestro 13.4.134[4]. The residues to be phosphorylated were modified using Maestro 13.4.134[4].

Simulation setup

The molecular dynamics simulations were performed using GROMACS 2021.2[5] , along with the AMBER ff99SB-ILDN force field[6]. The ATP molecule was modelled using the GAFF force field [7]. The Antechamber AMBER utility [8] and ACPYPE[9] were used to generate a GROMACS compatible topology. The integration time step used was 2 fs. The systems were simulated using periodic boundary conditions and at constant temperature and pressure. A cut-off of 1.0 nm was used for the van der Waals interactions. Long range electrostatic were treated using the particle mesh Ewald (PME) method using a real-space cut-off of 1.0 nm[10, 11]. neighbour lists were updated every 40 steps. The TIP3P model for water was used[12]. The temperature was set at 310 K, using a velocity rescale thermostat[13] and the pressure was set at 1 atm using an isotropic coupling with a Parrinello-Rahman bath[14].

The protein-ATP-Mg^{2+} complexes were solvated, and ions were added until system neutrality was achieved. The potential energy of the system was minimized in two steps, using a maximum of 50000 steps each: the first one, using position restraints of 1000 kJ/mol in all the heavy atoms, and the second stage with position restraints of the same magnitude in the protein–ligand complex heavy atoms only. The equilibration phase as performed in three stages: the first at constant temperature for 100 ps with position restraints of 1000 kJ/mol in the protein and ligand heavy atoms. Temperature was controlled using a Berendsen thermostat[15]. The second equilibration stage was simulated at constant temperature and pressure for 1 ns with position restraints of 1000 kJ/mol on the protein C-alpha atoms only. Pressure was controlled using a Berendsen barostat with an isotropic coupling [15]. Temperature was controlled using a velocity rescale thermostat[13]. Finally, the last stage of equilibration lasted for 1 ns with no position restraints. Temperature was controlled was controlled using a velocity rescale thermostat, while the pressure was controlled using an isotropic coupling with a Parrinello-Rahman bath[14].

Three replicates of each system (with no phosphorylation, double phosphorylated and triple phosphorylated) for both mutant and wild type by assigning different initial velocities in the beginning of the equilibration stage. The systems were simulated for 300 ns each and the simulations were extended as needed to obtain 150 ns of equilibrated trajectory. Considering that the MEK1 contains a substantial number of disordered residues, only the rigid protein core, comprising residues 41 to 257 and residues 331 to 378, was considered to monitor simulation equilibrium.

Analysis

Analyses were done using the tools included in the GROMACS 2021.2 simulation package[5].

The harmonic ensemble similarity (DHES) is a measure of the similarity between ensembles, was derived by Lindorff-Larsen et al[16]. We have used the implementation available in the ENCORE toolkit62 included in the MDAnalysis package[17, 18]. DHES was calculated between different states using the C-α positions of each individual residue. The trajectories were previously fitted to a common structure and concatenated, so that the whole concatenated trajectories were compared. The secondary structure was determined using the DSSP program. The elements of secondary structure considered were α-helix, β-sheet, β-bridge, turn, and 3_{10} helix, according to the DSSP classification[19].

The clustering analysis were done using PCA (principal components analysis) based clustering, implemented in the gmx_clusterByFeatures tool[20]. The features used for clustering were the projection of the atomic coordinates on the eigenvectors. The number of eigenvectors used for projecting the trajectories were such that they allowed to capture at least 80% of the total variance. Afterwards, k-means clustering was used to obtain conformational clusters, based on the RMSD (root mean square deviation) between central structures of each cluster. A structure was considered to be a part of a cluster if its RMSD is lower than 0.1 in comparison to the central one. All the protein C-alpha atoms were used in this process.

The images referring to structures were produces using NGL viewer version 2.0.0 and PyMOL version 1.8[21]. The error bars correspond to the 95% confidence interval obtained by bootstrapping. The plots were produced using Matplotlib package in Python[22].

Supplementary Note References

1. Jumper, J., et al., *Highly accurate protein structure prediction with AlphaFold.* Nature, 2021. **596**(7873): p. 583-589.
2. Fischmann, T.O., et al., *Crystal Structures of MEK1 Binary and Ternary Complexes with Nucleotides and Inhibitors.* Biochemistry, 2009. **48**(12): p. 2661-2674.
3. Olsson, M.H.M., et al., *PROPKA3: Consistent Treatment of Internal and Surface Residues in Empirical pKa Predictions.* Journal of Chemical Theory and Computation, 2011. **7**(2): p. 525-537.
4. Schrödinger, L., New York, NY, *Schrödinger Release 2023-1: Maestro, Schrödinger,LLC*. 2021.
5. Abraham, M.J., et al., *GROMACS: High performance molecular simulations through multi-level parallelism from laptops to supercomputers.* SoftwareX, 2015. **1-2**: p. 19-25.

6. Lindorff-Larsen, K., et al., *Improved side-chain torsion potentials for the Amber ff99SB protein force field.* Proteins: Structure, Function, and Bioinformatics, 2010. **78**(8): p. 1950-1958.
7. Wang, J., et al., *Development and testing of a general amber force field.* Journal of Computational Chemistry, 2004. **25**(9): p. 1157-1174.
8. Wang, J., et al., *ANTECHAMBER: an accessory software package for molecular mechanical calculations.* Journal of Chemical Information and Computer Sciences - JCISD, 2000. **222**.
9. Sousa da Silva, A.W. and W.F. Vranken, *ACPYPE - AnteChamber PYthon Parser interfacE.* BMC Research Notes, 2012. **5**(1): p. 367.
10. Petersen, H.G., *Accuracy and efficiency of the particle mesh Ewald method.* The Journal of Chemical Physics, 1995. **103**(9): p. 3668-3679.
11. Darden, T., D. York, and L. Pedersen, *Particle mesh Ewald: An N·log(N) method for Ewald sums in large systems.* The Journal of Chemical Physics, 1993. **98**(12): p. 10089-10092.
12. Mark, P. and L. Nilsson, *Structure and Dynamics of the TIP3P, SPC, and SPC/E Water Models at 298 K.* The Journal of Physical Chemistry A, 2001. **105**(43): p. 9954-9960.
13. Bussi, G., D. Donadio, and M. Parrinello, *Canonical sampling through velocity rescaling.* The Journal of Chemical Physics, 2007. **126**(1): p. 014101.
14. Parrinello, M. and A. Rahman, *Polymorphic transitions in single crystals: A new molecular dynamics method.* Journal of Applied Physics, 1981. **52**(12): p. 7182-7190.
15. Berendsen, H.J.C., et al., *Molecular dynamics with coupling to an external bath.* The Journal of Chemical Physics, 1984. **81**(8): p. 3684-3690.
16. Tiberti, M., et al., *ENCORE: Software for Quantitative Ensemble Comparison.* PLOS Computational Biology, 2015. **11**(10): p. e1004415.
17. Michaud-Agrawal, N., et al., *MDAnalysis: A toolkit for the analysis of molecular dynamics simulations.* Journal of Computational Chemistry, 2011. **32**(10): p. 2319-2327.
18. Gowers, R., et al., *MDAnalysis: a Python package for the rapid analysis of molecular dynamics simulations*. 2016.
19. Kabsch, W. and C. Sander, *Dictionary of protein secondary structure: Pattern recognition of hydrogen-bonded and geometrical features.* Biopolymers, 1983. **22**(12): p. 2577-2637.
20. Kumar, R., *gmx_clusterByFeatures* 2018.
21. LLC., S., *The PyMOL Molecular Graphics System*. 2015.
22. Hunter, J., *Matplotlib: A 2D Graphics Environment.* Computing in Science & Engineering, 2007. **9**: p. 90-95.
