# Supplementary material for: The identification of functional regions of MEK1 using CRISPR tiling screens
Source: Commun Biol. 2025 Apr 24;8:656. doi: 10.1038/s42003-025-07966-4 (PMC12022096; doi:10.1038/s42003-025-07966-4)
Supplement: Supplementary file 2 — Description of Additional Supplementary File [file 42003_2025_7966_MOESM2_ESM.docx]

**Description of Additional Supplementary File**

File name: **Supplementary Data 1.**

Description: **MEK1 sgRNA library sequences and annotations.** Sequences of sgRNAs added to the original library (Donovan et al., 2017) are highlighted in red.

File name: **Supplementary Data 2.**

Description: **CRISPRO output for drop-out screen using PAR/Cas9-D14 sgRNA LFC values.** LFC values in Supplementary Data 7 were used as input for CRISPRO. “rep1_LFC”, replicate 1 LFC values; “rep2_LFC”, replicate 2 LFC values. Mean of the LFC values (“reps_LFC_avg”) were added in the output and sorted from smallest to largest.

File name: **Supplementary Data 3.**

Description: **CRISPRO output with LFC values corrected to sgRNA on-target activity.** “norm_crispron”: drop-out screen sgRNA LFC values corrected to CRISPRon.

File name: **Supplementary Data 4.**

Description: **Prediction summary of MEK1 3EQI by Fpocket.** Output file by Fpocket indicating properties for all predicted pockets. “Pocket #”: number of predicted pockets. “Score”: overall pocket score. “Druggability Score”: predicted druggability score.

File name: **Supplementary Data 5.**

Description: **NGS results of MEK1 mutations generated by sgR234/L235 and analysis.** Data processing steps are provided. “Day1_rep1”, “Day9_rep1”, “Day1_rep2” and “Day1_rep2” sheets are the CRISPResso outputs “Alleles_frequency_table”, in which RPMs of each MEK1 mutation were calculated and added. LFC values of the in-frame mutations were calculated and sorted in sheets: “Rep1_inframe>0.02%” and “Rep2_inframe>0.02%”. Most depleted and enriched mutations were ranked in sheets: “rep1_selected” and “rep2_selected”.

File name: **Supplementary Data 6.**

Description: **LFC of frameshift and in-frame INDEL mutations generated by sgR234/L235.** Mutants generated by sgR234/L235 in two replicates were listed on separate data sheets. Mutants listed in the first column. “day2_day1”, “day5_day1” and “day9_day1”: LFC values of the same mutant on day 2, day 5 and day9 calculated towards day 1. “Class”: whether the mutant is frameshift or in-frame.

File name: **Supplementary Data 7.**

Description: **CRISPRO output for the enrichment screens.** LFC values in Supplementary Data 8 were used as input for CRISPRO. “sel_rep1_LFC”, replicate 1 LFC values of screen with selumetinib; “tra_rep1_LFC”, replicate 1 LFC values of screen with trametinib; “cobi_rep1_LFC”, replicate 1 LFC values of screen with cobimetinib; “bini_rep1_LFC”, replicate 1 LFC values of screen with binimetinib. Mean of LFC values for screens with each MEKi (“sel_LFC_avg”, “tra_LFC_avg”, “cobi_LFC_avg” and “bini_LFC_avg”) were added in the output. “sel_LFC_avg” was sorted from smallest to largest.

File name: **Supplementary Data 8.**

Description: **Complete CRISPR tiling screen datasets with LFC values.** NGS raw reads were counted and transformed to RPMs. LFC values were calculated between different cell lines and different days of the tiling screens. “SupData5_PARPARLFCvalues”, PAR/PAR LFC values of dropout screen; “SupData6_Cas9Cas9LFCvalues”, Cas9/Cas9 LFC values of dropout screen; “SupData7_PARCas9LFCvalues”, PAR/Cas9 LFC values of dropout screen; “SupData8_EnrichScreenLFCvalues”, LFC values of enrichment screens with four MEKi.

File name: **Supplementary Data 9-14.**

Description: **AlphaFold homology models.** Homology models of MEK1 wild type (Supplementary Data 9), R234_L235del variant (Supplementary Data 10), Q236_G237del variant (Supplementary Data 11), G237V variant (Supplementary Data 12), I141_C142delinsEI variant (Supplementary Data 13) and A372del variant (Supplementary Data 14) with the highest confidence (ranked_0.pdb files), which were used for structural analysis and MD simulation are provided.

File name: **Supplementary Data 15.**

Description: **CRISPRO output guide density.** Overview of sgRNAs targeting MEK1 with average density covering the residues.

File name: **Supplementary Data 16-18.**

Description: Source data for graphs.
